# Investigation of autism-related transcription factors underlying sex differences in the effects of bisphenol A on transcriptome profiles and synaptogenesis in the offspring hippocampus

**DOI:** 10.1186/s13293-023-00496-w

**Published:** 2023-02-20

**Authors:** Surangrat Thongkorn, Songphon Kanlayaprasit, Kasidit Kasitipradit, Pattanachat Lertpeerapan, Pawinee Panjabud, Valerie W. Hu, Depicha Jindatip, Tewarit Sarachana

**Affiliations:** 1 Department of Clinical Chemistry, Faculty of Allied Health Sciences, Chulalongkorn University, Bangkok, Thailand; 2 SYstems Neuroscience of Autism and PSychiatric Disorders (SYNAPS) Research Unit, Department of Clinical Chemistry, Faculty of Allied Health Sciences, Chulalongkorn University, 154 Soi Chula 12, Rama 1 Road, Wangmai, Pathumwan, Bangkok, 10330 Thailand; 3 Department of Biochemistry and Molecular Medicine, The George Washington University School of Medicine and Health Sciences, The George Washington University, Washington, DC USA; 4 Department of Anatomy, Faculty of Medicine, Chulalongkorn University, Bangkok, Thailand

**Keywords:** Sex difference, Endocrine-disrupting chemical, Bisphenol A, Transcription factor, Molecular docking, Androgen receptor, Synaptogenesis, Autism spectrum disorder, Microplastics

## Abstract

**Background:**

Bisphenol A (BPA) has been linked to susceptibility to autism spectrum disorder (ASD). Our recent studies have shown that prenatal BPA exposure disrupted ASD-related gene expression in the hippocampus, neurological functions, and behaviors associated with ASD in a sex-specific pattern. However, the molecular mechanisms underlying the effects of BPA are still unclear.

**Methods:**

Transcriptome data mining and molecular docking analyses were performed to identify ASD-related transcription factors (TFs) and their target genes underlying the sex-specific effects of prenatal BPA exposure. Gene ontology analysis was conducted to predict biological functions associated with these genes. The expression levels of ASD-related TFs and targets in the hippocampus of rat pups prenatally exposed to BPA were measured using qRT-PCR analysis. The role of the androgen receptor (AR) in BPA-mediated regulation of ASD candidate genes was investigated using a human neuronal cell line stably transfected with AR-expression or control plasmid. Synaptogenesis, which is a function associated with genes transcriptionally regulated by ASD-related TFs, was assessed using primary hippocampal neurons isolated from male and female rat pups prenatally exposed to BPA.

**Results:**

We found that there was a sex difference in ASD-related TFs underlying the effects of prenatal BPA exposure on the transcriptome profiles of the offspring hippocampus. In addition to the known BPA targets AR and ESR1, BPA could directly interact with novel targets (i.e., KDM5B, SMAD4, and TCF7L2). The targets of these TFs were also associated with ASD. Prenatal BPA exposure disrupted the expression of ASD-related TFs and targets in the offspring hippocampus in a sex-dependent manner. Moreover, AR was involved in the BPA-mediated dysregulation of *AUTS2*, *KMT2C*, and *SMARCC2*. Prenatal BPA exposure altered synaptogenesis by increasing synaptic protein levels in males but not in females, but the number of excitatory synapses was increased in female primary neurons only.

**Conclusions:**

Our findings suggest that AR and other ASD-related TFs are involved in sex differences in the effects of prenatal BPA exposure on transcriptome profiles and synaptogenesis in the offspring hippocampus. These TFs may play an essential role in an increased ASD susceptibility associated with endocrine-disrupting chemicals, particularly BPA, and the male bias of ASD.

**Supplementary Information:**

The online version contains supplementary material available at 10.1186/s13293-023-00496-w.

## Highlights

- Prenatal BPA exposure altered the transcriptome profiles of genes associated with ASD in the offspring hippocampus through several ASD-related transcription factors, including AR and ESR1, in a sex-dependent manner.
- In addition to the known BPA targets (i.e., AR and ESR1), other ASD-related transcription factors, including KDM5B, SMAD4, and TCF7L2, are also involved in the BPA effects in the offspring hippocampus, possibly by direct interactions with BPA.
- Prenatal BPA exposure also caused a significant reduction in the expression of ASD-related TFs in the hippocampus of female offspring but not in that of males.
- ASD candidate genes (i.e., *AUTS2*, *SMARCC2*, and *KMT2C*), which are known AR targets, were differentially expressed in response to BPA in the human neuronal cell line with AR overexpression compared to the cell line with low AR protein levels, indicating that AR is involved in BPA-mediated effects on these ASD candidate genes.
- BPA-responsive genes that are transcriptional targets of AR and other ASD-related transcription factors are associated with synaptogenesis. Prenatal BPA exposure caused an increase in synaptic protein levels in primary hippocampal neurons and in hippocampal tissues of male offspring but not in females.

**Supplementary Information:**

The online version contains supplementary material available at 10.1186/s13293-023-00496-w.

## Introduction

Bisphenol A (BPA) is an endocrine-disrupting chemical (EDC) that is widely used in polycarbonate plastics and epoxy resins. BPA can be found in several consumable products used in daily life, including the linings of food and beverage containers, dental sealants, thermal paper receipts, and recycled toilet paper [1, 2]. Moreover, BPA can be found in micro/nanoplastics, which have become a major environmental problem around the world [3]. In humans, the most common route of BPA exposure is oral ingestion, but BPA exposure can also occur through dermal contact or inhalation. Recent studies have shown that BPA can be detected in the serum and urine of pregnant women and children [4, 5]. BPA can cross the placenta [6] and the blood–brain barrier [7]. Exposure to BPA disrupts brain developmental processes, including neurogenesis [8], neuron migration [9], neurite outgrowth [10], and synaptogenesis [11, 12]. Our recent studies revealed that BPA is an environmental risk factor for neurological disorders, including autism spectrum disorder (ASD) [10, 13, 14] and Alzheimer's disease (AD) [15].

ASD is an early-onset neurodevelopmental disorder that is characterized by two main symptoms: (1) social interaction and communication deficits and (2) repetitive behaviors and restricted interests. ASD is a significant public health problem worldwide, with a prevalence of 1 in 44 children in the United States, as reported by the Centers for Disease Control and Prevention (CDC) [16]. ASD is a male-biased disorder that is at least four times more common in males than in females [17]. The exact cause of ASD is still unclear, but it is thought that both genetic and environmental factors influence ASD [10, 13, 14, 18–22]. Moreover, recent studies have reported that epigenetic regulatory mechanisms, including DNA methylation [19, 20, 22], histone modifications [23, 24], and noncoding RNA-associated gene silencing [25], are associated with ASD. Environmental factors that are thought to cause or increase the susceptibility of ASD include BPA and bisphenol-related compounds [10, 13, 14, 18, 26–28], polychlorinated biphenyls (PCBs), phthalates [29, 30], and cigarette smoke [31]. BPA can be detected in the serum and urine of individuals with ASD [26–28]. Kardas et al. investigated the serum BPA levels of children with ASD using high-performance liquid chromatography (HPLC) and reported that serum BPA was elevated in children with ASD compared to healthy control children [26]. In addition, Kondolot et al. measured BPA levels in ASD children and found a significant increase in plasma BPA levels in children with pervasive developmental disorder not otherwise specified (PDD-NOS), which is a subtype of ASD, compared to typically developing children [28]. Moreover, Hansen et al. measured BPA levels in maternal urine samples collected in gestational week 28 and assessed ASD symptoms of the children at the ages of 2 and 5 years [5]. Elevated odds ratios were observed among 5-year-old children within the 3rd tertile of BPA exposure with an ASD score above the 75th percentile, suggesting that prenatal BPA exposure may increase the risk of ASD symptoms [5]. These findings support that BPA exposure is a risk factor contributing to ASD.

Recent studies have reported that exposure to BPA disrupts the expression of genes in several brain regions, including the hippocampus [10, 13, 32], prefrontal cortex [14], hypothalamus [32], cerebellum [33], and amygdala [34]. The hippocampus is a brain region that plays an important role in learning and memory and is one of the brain regions known to be negatively impacted in ASD [35, 36]. Arambula et al. studied the effects of prenatal BPA exposure on the transcriptome profiles of the hypothalamus and hippocampus of neonatal rat offspring and found that prenatal BPA exposure induced sex-specific changes in *Esr1* and *Esr2* expression in the hypothalamus and oxytocin (*Oxt*) in the hippocampus [32]. Moreover, exposure to BPA during pregnancy caused apoptosis of neurons in the hippocampus of offspring [37]. Both the alterations in the expression of these genes and the apoptosis of hippocampal neurons induced by prenatal BPA exposure have been demonstrated to be involved in susceptibility to ASD [38–40].

Recently, we performed transcriptome profiling of hippocampal tissues isolated from neonatal rat pups prenatally exposed to BPA using RNA-seq analysis and found that prenatal BPA exposure caused dysregulation of genes in the hippocampus of offspring in a sex-dependent manner [10, 13]. Significantly differentially expressed genes (DEGs) in male and female rat pups showed a significant association with ASD, with a stronger association in the male group than in the female group [13]. In addition, we investigated the effects of prenatal BPA exposure on ASD-related genes (i.e., *Arhgap32, Atp8a1, Cux1*, *Eif3h*, *Itga4*, *Kdm5c*, and *Mief2*) known to regulate neuronal viability, neuritogenesis, and learning/memory and assessed these functions in the offspring. We found that the expression of these genes showed a male-specific relationship with abnormal neurological functions [10], suggesting that these genes may play an important role in BPA-induced adverse effects on hippocampus-related functions and on the learning/memory of male pups.

In addition to neuronal viability and neuritogenesis, exposure to BPA has been reported to affect synaptogenesis [11, 12, 41, 42]. Several studies have investigated the effects of BPA on adult synapse density [41, 42], but the effects of prenatal BPA exposure on synaptogenesis in the offspring brain remain unclear. Zhang et al. investigated the effects of prenatal BPA exposure on synaptogenesis in the offspring hippocampus and found that exposure to BPA decreased spine density in the hippocampus of the offspring [12]. Kawato et al. reported that perinatal exposure to BPA at 30 µg/kg·body weight of the mother reduced dendritic spine density in male offspring [11]. In female offspring, however, the total spine density was significantly increased at the estrus stage but decreased at the proestrus stage [11]. These findings indicate that there is a sex difference in the effects of prenatal BPA exposure on transcriptome profiles of genes associated with ASD and on neurological functions in the offspring hippocampus. However, the molecular mechanisms through which BPA exerts its sex-specific effects on the transcriptome profiles in the hippocampus of the offspring are still unclear.

In this study, we, therefore, sought to identify potential transcription factors that may be responsible for the sex difference in the effects of prenatal BPA exposure on the transcriptome profiles of ASD candidate genes in the hippocampus of offspring. First, we obtained transcriptome profiling data from RNA-seq analysis of hippocampal tissues isolated from neonatal rat pups prenatally exposed to BPA or vehicle control and identified differentially expressed genes (DEGs) in response to prenatal BPA exposure. Next, to identify transcription factors that may serve as upstream regulators of these DEGs, we obtained a list of transcription factors that have been associated with ASD and the targets of each ASD-related transcription factor. Then, hypergeometric distribution analysis was performed to identify transcription factors, whose transcriptional targets were significantly enriched in the list of BPA-responsive DEGs in the offspring hippocampus. Moreover, to determine whether BPA can directly interact with these ASD-related transcription factors, molecular docking analysis was performed using three-dimensional structures of BPA and these transcription factor molecules.

Since androgen receptor protein (AR) is one of the transcription factors known to interact with BPA and is thought to play an important role in the sex difference in the expression of ASD candidate genes [43–49], we further investigated whether AR is involved in the effects of BPA on the expression of ASD candidate genes. The human neuroblastoma cell line SH-SY5Y with low expression of AR protein was stably transfected with AR-overexpression plasmid or control plasmid and then treated with BPA or vehicle control. The expression of AR transcriptional targets which are also known ASD candidate genes was assessed using qRT-PCR analysis.

To further investigate biological functions, diseases, and pathways associated with the BPA-responsive DEGs that are the targets of ASD-related transcription factors, gene ontology analysis was performed using Ingenuity Pathway Analysis (IPA) software. As synaptogenesis was predicted to be one of the biological functions significantly associated with these DEGs, we further investigated the effects of prenatal BPA exposure on the synaptogenesis of hippocampal neurons isolated from male and female rat pups.

## Methods

### Collection of transcriptome profile data and the lists of DEGs in response to prenatal BPA exposure

RNA-seq data from our transcriptome profiling analysis of hippocampal tissues isolated from rat offspring prenatally exposed to BPA (*n* = 6 pups from independent litters; 3 males and 3 females) or vehicle control (*n* = 6 pups from independent litters; 3 males and 3 females) were obtained from the NCBI GEO Data sets database (accession number GSE140298) [13]. The details of animal husbandry, BPA treatment, and RNA-seq analysis were described in a previously published study [13]. Briefly, BPA was dissolved in molecular biology grade absolute ethanol and further diluted with corn oil to a final concentration of 5000 µg/kg·maternal body weight of BPA to treat each rat. The vehicle control treatment was prepared by mixing absolute ethanol with corn oil in amounts equivalent to those used for preparing BPA. After mating, each female rat was intragastrically administered either BPA or the vehicle control daily from GD1 until parturition. Total RNA was isolated from the hippocampus, and RNA-seq analysis was performed by BGI Genomics Co., Ltd using the Illumina HiSeq 4000 next-generation sequencing platform with 4 G reads (Illumina, Inc.) as previously described [13]. Poisson distribution analysis was performed to identify DEGs in response to BPA. Comparisons were performed with all male and female pups with the same treatment condition combined into one group and separately for each sex. DEGs with a *p* value < 0.05 and FDR < 0.05 were considered statistically significant.

### Identification of TFs that are upstream regulators of DEGs

To identify transcription factors that have been associated with ASD and may serve as the upstream regulators of these DEGs, we obtained the list of human TFs from the Human Transcription Factors database (http://humantfs.ccbr.utoronto.ca/allTFs.php) [50] and overlapped them with the list of genes associated with ASD from the SFARI database (https://gene-archive.sfari.org/database/human-gene/) [51] using Venny 2.1 software (https://bioinfogp.cnb.csic.es/tools/venny/) [52]. Transcriptional targets of these ASD-related transcription factors were predicted using the TRANSFAC Curated Transcription Factor Targets database [53, 54], the TRANSFAC Predicted Transcription Factor Targets database [53, 54], the JASPAR Predicted Transcription Factor Targets database [55, 56], the CHEA Transcription Factor Targets database [57], the ENCODE Transcription Factor Targets database [58, 59], and the MotifMap Predicted Transcription Factor Targets database [60] through the Harmonizome database [61]. To identify TFs whose transcriptional targets were significantly enriched in the lists of DEGs, hypergeometric distribution analysis between the list of DEGs in response to prenatal BPA exposure and the list of transcriptional targets of each TF was then performed using the Keisan Online Calculator package (http://keisan.casio.com/exec/system/1180573201) [62]. A *q* value < 0.05 was considered a significant association.

### Prediction of biological functions, disorders, and pathways associated with DEGs that are regulated by ASD-related TFs

Biological functions, disorders, and pathways associated with DEGs that were transcriptional targets of ASD-related TFs were predicted using Ingenuity Pathway Analysis (IPA) software (QIAGEN Inc., Hilden, Germany, https://www.qiagenbioinformatics.com/products/ingenuity-pathway-analysis/) [63]. The list of DEGs overlapped with the list of genes experimentally validated to be associated with each function/disorder/canonical pathway in the Ingenuity Knowledge Base database. Fisher’s exact test was then performed to calculate *p* values, and a *p* value < 0.05 was considered statistically significant.

### Expression of ASD-related TFs in the developing hippocampus

To assess the expression of ASD-related TFs in the developing hippocampus, the expression profile data of each TF in the hippocampus of mice at embryonic days 11.5, 13.5, 15.5, 18.5, and postnatal day (PND) 4 were obtained from the Allen Brain Atlas database (https://mouse.brain-map.org/) [64]. For rat offspring (PND1), the expression of ASD-related TFs in the hippocampus was also investigated using qRT-PCR analyses.

### In silico virtual screening of ASD-related TFs that are potential BPA targets

To predict whether BPA can directly interact with ASD-related TFs whose target genes are significantly differentially expressed in the hippocampus of rat offspring prenatally exposed to BPA, molecular docking analysis of BPA and those TFs was performed. For TFs whose ligands have been identified, molecular docking analysis of TFs and their known ligand(s) was also conducted for comparison with BPA. The three-dimensional structures of BPA and known ligands of each TF were obtained from the PubChem database (https://pubchem.ncbi.nlm.nih.gov/) [65] and processed to generate pdb files with clean geometry using BIOVIA Discovery Studio Visualizer 2020 [66, 67]. The processed BPA and ligand structures were then converted to pdbqt files for molecular docking analysis. For TFs, the three-dimensional structures of TFs were retrieved from the RCSB PDB Protein Data Bank database (https://www.rcsb.org/) [68]. TF structures obtained by the X-ray diffraction method with a resolution of less than 3 Å were selected for molecular docking analysis. Structures of water or other molecules surrounding TFs were removed, and all missing hydrogens and Kollman charges were added to the TF structure using AutoDockTools 4.2 software [69]. The processed TF structures were then converted to pdbqt files. Molecular docking analysis was performed as previously described [14]. Briefly, rigid docking of the TFs with BPA and their known ligands was performed using AutoDockTools 4.2 software with the Lamarckian GA algorithm and default parameters recommended by the software. For each run, a total of ten conformations of the BPA/ligand–TF complex were generated, and the conformation with the lowest binding free energy was selected. The mean and standard deviation values of the lowest binding-free energies from three independent runs were calculated and reported in this study. The interactions (i.e., hydrogen bonds and others) between each TF and BPA or known ligands were visualized using BIOVIA Discovery Studio Visualizer 2020 [66, 67].

### Animal husbandry and treatment

Eight-week-old male and female Wistar rats were obtained from the National Laboratory Animal Center (NLAC) in Thailand. All experimental procedures were approved by the Chulalongkorn University Animal Care and Use Committee (Animal Use Protocol No. 1673007, 1773011, and 2073011). All procedures were performed in accordance with relevant guidelines and regulations. All rats were housed at the Chulalongkorn University Laboratory Animal Center (CULAC) under standard temperature (21 ± 1 °C) and humidity (30–70%) conditions on a 12-h light/dark cycle with food and RO-UV water available ad libitum. Rats were treated as previously described in the transcriptome profiling study [10, 13–15]. Briefly, we synchronized the estrous cycle and stimulated the mating behaviors of female rats by adding the bedding from a male rat cage to the cage of female rats for 5 days prior to mating. After that, one male and one female were placed together in the same cage overnight. The presence of a vaginal plug was observed on the following day and considered gestational day 1 (GD1).

After mating, female rats were divided into two groups: the BPA treatment group and the vehicle control group. Each female rat was weighed daily to monitor pregnancy and calculate the dose of BPA or vehicle control and was intragastrically administered either BPA or vehicle control daily from GD1 until parturition. For BPA treatment, BPA (Sigma-Aldrich, USA) was dissolved in molecular biology grade absolute ethanol (Merck Millipore, USA) to a final concentration of 250 mg/mL and then further diluted with corn oil to a final concentration of 5000 µg/kg·maternal body weight of BPA to treat each rat. The vehicle control treatment was prepared by mixing molecular biology grade absolute ethanol (Merck Millipore, USA) with corn oil in amounts equivalent to those used for preparing BPA. The dose of BPA used in this study is equal to the No-Observed-Adverse-Effect Level (NOAEL) of BPA determined by the U.S. Food and Drug Administration [70]. To prevent cross-contamination of the treatment conditions, rats in the BPA and control groups were raised separately in individually ventilated cages in a biohazard containment housing system. Separate sets of stainless-steel needles and all consumable products were used for oral gavage. All reusable materials were cleaned with ethanol and rinsed with copious amounts of Milli-Q deionized water before use.

The sex of every neonatal rat pup was determined on PND1 by visual inspection of the external genitalia. Male and female pups were distinguished by the distance between the external genitalia and the anus of the pup by two trained researchers [71]. Male pups exhibited a greater distance between the external genitalia and the anus than females (Additional file 1). For hippocampus isolation, neonatal rat pups were anesthetized by intraperitoneal injection of 100 mg/kg·BW sodium pentobarbital and euthanized by decapitation on ice. The brain was immediately placed in ice-cold, freshly prepared dissection medium (Additional file 2), and the hippocampus was quickly dissected on ice under a Nikon SMZ18 Stereo Microscope (Nikon, Japan). Meninges were removed completely. The hippocampus was then stored in RNAlater (Ambion, USA) at − 80 °C for subsequent qRT-PCR analysis or placed in a prechilled 35-mm dish containing ice-cold dissection medium for isolation of primary hippocampal neurons.

### RNA isolation

Total RNA was extracted using the mirVana miRNA Isolation Kit (Ambion, USA) according to the manufacturer’s protocol. Briefly, hippocampal tissues from male and female rat pups (*n* = 3 pups/sex/treatment group from three different dams) were obtained and lysed in denaturing lysis buffer, which stabilized RNA and inactivated RNases. Total RNA was then extracted from the hippocampal tissue lysates using acid–phenol:chloroform and further purified over a glass–fiber filter. RNA was eluted from the purification column using the elution solution. The purity of total RNA was assessed using a NanoDrop spectrophotometer (Thermo Fisher Scientific, USA) and quantified using an Invitrogen Qubit 2.0 Fluorometer (Thermo Fisher Scientific, USA).

### Quantitative RT–PCR analysis

Purified total RNA samples isolated from the hippocampus of rat offspring prenatally exposed to BPA or vehicle control were reverse transcribed to cDNA using a Maxime RT PreMix Kit (iNtRON Biotechnology, South Korea) according to the manufacturer’s protocol. Briefly, total RNA (1 µg) was added to a Maxime RT PreMix tube, and distilled water was then added to the tube to a total volume of 20 µL. The cDNA synthesis reaction was performed by incubating the tube at 45 °C for 60 min, followed by 95 °C for 5 min. After incubation, cDNA was diluted with nuclease-free water to obtain a final concentration of 10 ng/µL and used as the template for subsequent qPCR analysis. Quantitative PCR analysis was conducted using RealMOD Green W 2X qPCR Master Mix (iNtRON Biotechnology, South Korea) according to the manufacturer’s protocol. Briefly, 1 µL of cDNA was mixed with RealMOD Green W 2 × qPCR mix, forward primer, reverse primer, and nuclease-free water. Each cDNA sample was run in triplicate reactions. qPCR was performed in a Bio-Rad CFX Connect Real-Time System (Bio-Rad, USA) using the following conditions: an initial activation step at 95 °C for 10 min, followed by 40 cycles of denaturing at 95 °C for 20 s and annealing/extension at 55 °C for 30 s. A no-template control was included for every gene to check for contamination in the reaction. A melting curve analysis was set from 65 to 95 °C to assess the specificity of primers and the formation of the PCR product. The expression levels were calculated by the 2^−ΔΔCt^ method using rat 18S ribosomal RNA (*Rn18s*) or human 18S ribosomal RNA (*RN18S*) as an endogenous control gene. Forward and reverse primers were designed for rat TFs (i.e., *Ar*, *Esr1*, *Egr2*, *Kdm5b*, *Sox5*, *Smad4*, *Tcf7l2*, and *Yy1*), rat DEGs (i.e., *Adnp*, *Chd2*, *Cic*, *Elavl2*, *Esrrb*, *Kat6a*, *Kmt2c*, *Nfib*, and *Snap25*), and human genes (i.e., *AUTS2*, *DICER1*, *ESR1*, *ESR2*, *KMT2C*, and *SMARCC2*). The sequences of all primers are shown in Additional file 3.

### Overexpression of AR in a human neuroblastoma cell line and BPA treatment

To investigate the role of AR in the effects of BPA on the expression of ASD candidate genes, the human neuroblastoma cell line SH-SY5Y (ATCC, USA) stably transfected with AR-expression plasmid or negative control plasmid was used. These cell lines were kindly provided by Associate Professor Ray-Chang Wu and Professor Valerie Hu, George Washington University, USA. For the AR-expression plasmid, the DNA sequence encoding the DNA binding and transcription activation domains of human AR was inserted into pSG5.HA expression vector containing a neomycin resistance gene. An empty pSG5.HA expression vector containing a neomycin resistance gene was used as a negative control vector. SH-SY5Y cells were stably transfected with the AR-expression plasmid or negative control plasmid using Lipofectamine LTX reagent with PLUS reagents (Invitrogen, USA) according to the manufacturer’s protocol. These cells were cultured in 1:1 MEM/EBSS with l-glutamine (GE Healthcare, USA) and Ham’s F12 media (GE Healthcare, USA) supplemented with 15% fetal bovine serum (Sigma-Aldrich, USA) and 1000 µg/mL geneticin (G418; Invitrogen, USA) in a 37 °C incubator with 5% CO_2_. Geneticin was added to the media to select only the cells containing the neomycin-resistant plasmid. Western blot analysis was conducted to assess the expression of AR protein in these stably transfected cells (Additional file 4).

For BPA treatment, SH-SY5Y cells stably transfected with AR-expression or negative control plasmid were trypsinized and washed with PBS (GE Healthcare, USA). Then, cells were resuspended in 1:1 phenol red-free DMEM/F12 media (Invitrogen, USA) supplemented with 15% fetal bovine serum stripped with charcoal–dextran (Invitrogen, USA) and 1000 µg/mL geneticin (G418, Invitrogen, USA), and seeded at 1.5 × 10^6^ cells per well in a 6-well plate precoated with 10 µg/cm^2^ collagen type I (Sigma-Aldrich, USA). Phenol red-free media and charcoal–dextran stripped serum were used in this experiment to minimize confounding effects from the phenol-related compound in the media and endogenous hormones in the serum. At 24 h after seeding, cells were induced to differentiate by replacing the medium with 1:1 phenol red-free DMEM/F12 medium (Invitrogen, USA) supplemented with 1% fetal bovine serum stripped with charcoal–dextran (Invitrogen, USA), 1,000 µg/ml geneticin (G418; Invitrogen, USA), and 10 µM retinoic acid (RA; Sigma-Aldrich, USA). BPA (Sigma-Aldrich, USA) was dissolved in molecular biology grade absolute ethanol (Merck Millipore, USA) to a final concentration of 250 mg/mL, and then added to the differentiation media to a final concentration of 1 ng/mL. For the vehicle control treatment, molecular biology grade absolute ethanol (Merck Millipore, USA) in amounts equivalent to those used for preparing BPA was added to the differentiation media. The cells were differentiated for 7 days and then lysed in GENEzol reagent (Geneaid Biotech, Taiwan) for RNA extraction. Total RNA was then isolated from the cell lysates using the phenol:chloroform method according to the manufacturer’s protocol. The purity and concentration of total RNA were assessed using a NanoDrop spectrophotometer (Thermo Fisher Scientific, USA).

### Synaptogenesis assay

Primary hippocampal neurons were isolated from neonatal rat pups that were prenatally exposed to BPA (5000 µg/kg·maternal BW per day) or vehicle control. Primary hippocampal neurons were cultured according to Beaudoin et al. (2012) with slight modifications [72]. Briefly, the hippocampus of neonatal rat pups on PND1 was dissected and kept in ice-cold, freshly prepared dissection medium (Additional file 2). Then, the medium was removed, and the tissues were digested using 5 ml 2.5% w/v trypsin solution (Thermo Fisher Scientific, USA) and incubated at 37 °C for 20 min. Then, 25 µl 1% DNase I solution (Sigma-Aldrich, USA) was added to the tube and incubated at room temperature for 5 min. The hippocampal tissue chunks were gently washed twice in ice-cold, freshly prepared dissection medium. Hippocampal tissues were resuspended in plating medium (Additional file 2) and gently triturated using a fire-polished glass Pasteur pipette to dissociate the cells. The cells were counted and seeded in a 35-mm cell culture dish containing a 22-mm coverslip precoated with poly-l-lysine (Sigma-Aldrich, USA) at 2 × 10^5^ cells/coverslip. The cells were allowed to attach to the coverslip for 4 h in a CO_2_ incubator. Then, the plating medium was gently removed and replaced with fresh maintenance medium (Additional file 2). After two days of incubation at 37 °C and 5% CO_2_, half of maintenance medium was replaced with the maintenance medium supplemented with cytosine arabinoside (ara-C, 1-b-d-arabinofuranosylcytosine; Sigma-Aldrich, USA) at a final concentration of 2 µM to inhibit the proliferation of nonneuronal cells. Cells were further incubated for a total of 14 days after seeding. Half of the maintenance medium was replaced with fresh maintenance medium without ara-C once every 3 days.

To investigate the effects of prenatal BPA treatment on synaptogenesis, mature primary hippocampal neurons (DIV14) were gently washed with ice-cold PBS and then fixed with freshly prepared 4% paraformaldehyde in PBS for 15 min. The cells were washed with PBS for 5 min three times and blocked using 3% BSA (Capricorn Scientific, Germany) at room temperature for 30 min in a dark chamber. The cells were then immunostained for synaptic markers, including Syn1 (presynaptic marker), Psd95 (postsynaptic marker), and Map2 (mature neuron marker). Rabbit anti-Syn1 (ab8; Abcam, UK), mouse anti-Psd95 (ab2723; Abcam, UK), and chicken anti-Map2 (ab5392; Abcam, UK) antibodies were added to the cells and incubated at 4 °C overnight. On the following day, donkey anti-rabbit Alexa 647 (ab150063; Abcam, UK), donkey anti-mouse Alexa 488 (ab150109; Abcam, UK), and donkey anti-chicken Alexa 405 (ab175675; Abcam, UK) secondary antibodies were added to the cells and incubated for 1 h at room temperature. The coverslip with cells was mounted to a glass slide using ProLong Diamond Antifade reagent (Invitrogen, USA). The experiment was performed using three litters of pups/sex/treatment group. For each litter of pups, a total of 15 differentiated primary hippocampal neurons were captured using a LSM800 confocal laser scanning microscope (Carl Zeiss, Germany). The fluorescence intensities of Syn1, Psd95, and Map2 staining were measured using ZEN Blue software (Carl Zeiss, Germany). The number of Syn1 and Psd95 colocalization sites on mature neurons was counted as synaptic puncta using Imaris software (Bitplane, UK).

### Western blot analysis

Western blot analysis was performed to investigate the expression of synaptic proteins in the hippocampus of rat offspring prenatally exposed to BPA and vehicle control. Hippocampal tissues from the pups in the BPA group (*n* = 6 pups; 3 males and 3 females from 3 different dams) and the vehicle control group (*n* = 6 pups; 3 males and 3 females from 3 different dams) were used. One male and one female pup were randomly selected from each dam. The hippocampal tissues were dissected from the rat pups (PND1), and protein was isolated using Genezol reagent (Geneaid Biotech, Taiwan) according to the manufacturer’s protocol. Protein pellets were resuspended in lysis buffer consisting of 7 M urea, 2 M thiourea, 4% w/v CHAPS, and 100 mM dithiothreitol (DTT). The concentrations of the protein samples were measured using Bradford protein assays using Bradford reagent (Bio-Rad, USA), with bovine serum albumin (BSA; Capricorn Scientific, Germany) as a standard. A total of 15 µg of each protein sample was separated via 10% SDS-PAGE and then transferred to 0.2 µm Immu-Blot PVDF membranes (Bio-Rad, USA) using a Mini-PROTEAN Tetra system (Bio-Rad, USA). The membrane was blocked with 5% nonfat dry milk (Bio-Rad, USA) in TBST for 1 h at room temperature. After that, the membrane was incubated overnight at 4 °C with rabbit anti-Syn1 antibody (1:10,000, 106011; Synaptic Systems, Germany), rabbit anti-Syp antibody (1:1,000, ab52636; Abcam, UK), mouse anti-Psd95 antibody (1:1,000, ab2723; Abcam, UK), and rabbit anti-Map2 antibody (1:1,000, #4542; Cell Signaling Technology, USA). The membrane was then washed and incubated with donkey anti-rabbit (ab7083, Abcam, UK) conjugated with HRP or donkey anti-mouse conjugated with HRP antibody (ab205724; Abcam, UK) for 1 h at room temperature. The membrane was visualized by Amersham ECL Western Blotting Detection Reagent (Cytiva, USA), and band images were taken using an Amersham ImageQuant 800 Western Blot Imaging System (Cytiva, USA). Band intensity was analyzed using ImageJ software [73].

### Prediction of TFs involved in the effects of prenatal BPA exposure on the transcriptome profiles of the offspring hippocampus using data from other independent studies

To determine whether the ASD-related TFs predicted to be BPA targets and regulate DEGs in the offspring hippocampus were reproducible when other transcriptome profiling data were used, transcriptome profiling data of hippocampal tissues from rats or mice exposed to BPA in utero from previously published studies of other groups were obtained from the NCBI PubMed database [74] in a search performed on September 12, 2022, using the keywords “bisphenol A” and “hippocampus.” Studies that met the following criteria were included in this study: (i) the animal model was rodents and exposed to BPA in utero; (ii) transcriptome profiling analysis of the hippocampus of offspring was performed using RNA-seq or microarray techniques; and (iii) raw RNA-seq or microarray data or the complete list of all DEGs in response to prenatal/perinatal BPA exposure in the hippocampus were provided. When raw transcriptome profiling data were available, the data were obtained and reanalyzed using the Galaxy platform (https://usegalaxy.org/) [75]. DEGs were then identified by DESeq2 analysis, and *p*-value < 0.05 and FDR < 0.05 were considered statistically significant. If raw transcriptome data were not available, complete lists of all DEGs reported in the study were obtained. Hypergeometric distribution analysis was then performed to determine the association between the list of DEGs from these studies and the list of transcriptional targets of ASD-related TFs.

### Statistical analyses

The statistical analyses were performed using the SPSS software package for Windows [76]. Two-tailed Student’s *t* test analysis was performed to determine the statistical significance of the differences in the mean values. A *p* value < 0.05 was considered statistically significant.

## Results

### Dysregulated genes in the hippocampus of offspring prenatally exposed to BPA were transcriptional targets of ASD-related TFs

Our recent studies have shown that prenatal BPA exposure altered the transcriptome profiles in the hippocampus of offspring, and changes in the expression of ASD candidate genes in response to BPA showed a male-specific correlation with hippocampal functions [10, 13]. Moreover, we found that BPA-responsive genes in the prefrontal cortex of rat offspring were potentially regulated by several ASD-related TFs, including AR, ESR1, and RORA [14]. To determine whether genes that were differentially expressed in the hippocampus of offspring prenatally exposed to BPA were transcriptional targets of ASD-related TFs, we obtained lists of DEGs from our previous RNA-seq analysis of hippocampal tissues isolated from rat offspring prenatally exposed to BPA (*n* = 6 pups; 3 males and 3 females) or vehicle control (*n* = 6 pups; 3 males and 3 females) for subsequent analysis. When both male and female pups in the same treatment group were combined into one group, a total of 4525 genes were reported to be significantly dysregulated in response to prenatal BPA exposure [13]. When each sex of the pups was analyzed separately, a total of 2078 and 3055 genes were found to be differentially expressed in the male and female groups, respectively [13]. These lists of DEGs were used for subsequent analysis in this study.

To identify transcription factors that have been associated with ASD and may serve as upstream regulators of these DEGs, we obtained a list of 1639 proteins identified as transcription factor proteins from the Human Transcription Factors database. Among these TFs, a total of 96 TFs have been determined to be ASD candidate genes/proteins by the SFARI database. The list of these ASD-related TFs is shown in Additional file 5. Since TFs are known to regulate the transcription of multiple genes, we obtained the list of transcriptional targets of each ASD-related TF from multiple databases through the Harmonizome database. The transcriptional targets of 34 ASD-related TFs have been identified and provided in the Harmonizome database.

To predict whether the DEGs in the hippocampus of offspring prenatally exposed to BPA were regulated by ASD-related TFs, the lists of DEGs and the list of transcriptional targets of each ASD-related TF were compared. Hypergeometric distribution analysis was then performed to assess the significance of the association between DEGs and transcriptional targets of each TF (Additional file 6). The hypergeometric distribution analysis revealed eight ASD-related TFs whose transcriptional targets were significantly enriched in the lists of DEGs (*q* value < 0.05; Table 1). These TFs were AR, ESR1, EGR2, KDM5B, SOX5, SMAD4, TCF7L2, and YY1. Interestingly, the targets of seven TFs (i.e., AR, ESR1, EGR2, KDM5B, SOX5, SMAD4, and YY1) were significantly associated with BPA-responsive genes in a sex-dependent pattern. The targets of AR, ESR1, EGR2, KDM5B, and SOX5 were manually curated by the TRANSFAC Curated Transcription Factor Targets database. This finding suggests that these ASD-related TFs may serve as potential upstream regulators of BPA-responsive genes, and BPA may disrupt the transcriptome profiles in the hippocampus of male and female offspring through different ASD-related TFs.

**Table 1:** Hypergeometric distribution analysis of transcriptional targets of ASD-related TFs and BPA-responsive genes in the hippocampus of male and female offspring

	Male (2078 DEGs)			Female (3055 DEGs)			Both sexes (4525 DEGs)		
TFs	TRANSFAC Curated *p* value (#overlapping genes/#targets)	TRANSFAC Predicted *p* value (#overlapping genes/#targets)	CHEA *p* value (#overlapping genes/#targets)	TRANSFAC Curated *p* value (#overlapping genes/#targets)	TRANSFAC Predicted *p* value (#overlapping genes/#targets)	CHEA *p* value (#overlapping genes/#targets)	TRANSFAC Curated *p* value (#overlapping genes/#targets)	TRANSFAC Predicted *p* value (#overlapping genes/#targets)	CHEA *p* value (#overlapping genes/#targets)
AR	**0.038 (96/755)**	NA	0.999 (595/6279)	0.574 (135/755)	NA	0.999 (1011/6279)	0.994 (179/755)	NA	0.999 (1536/6279)
ESR1	**0.027 (63/465)**	NA	0.692 (234/2253)	0.426 (86/465)	NA	0.235 (420/2253)	0.854 (119/465)	NA	0.730 (611/2253)
EGR2	0.560 (20/190)	NA	NA	**0.044 (44/190)**	NA	NA	0.685 (50/190)	NA	NA
KDM5B	NA	NA	**2.06E−07 (463/3555)**	NA	NA	**2.90E−05 (725/3555)**	NA	NA	0.985 (932/3555)
SOX5	**6.16E−04 (45/258)**	NA	NA	**0.029 (59/258)**	NA	NA	0.892 (63/258)	NA	NA
SMAD4	0.435 (26/233)	**0.033 (338/2904)**	0.125 (493/4430)	0.337 (45/233)	0.072 (553/2,904)	0.890 (775/4430)	0.997 (47/233)	0.999 (703/2904)	0.999 (1140/4430)
TCF7L2	NA	NA	0.826 (54/565)	NA	NA	0.311 (107/565)	NA	NA	**0.040 (175/565)**
YY1	**0.020 (97/744)**	0.961 (151/1,602)	0.660 (260/2488)	0.087 (149/744)	0.970 (263/1602)	0.997 (402/2488)	0.986 (180/744)	0.999 (391/1602)	0.999 (598/2488)

Values in bold represent statistically significant results

Transcriptional targets of each TF were obtained from the TRANSFAC Curated, the TRANSFAC Predicted, and the CHEA databases through the Harmonizome database. The lists of DEGs in the hippocampus of male and female pups prenatally exposed to BPA were obtained from our previously published transcriptome profiling study [13]. A *p* value < 0.05 was considered significant

*NA* not applicable

### BPA-responsive genes that are transcriptional targets of ASD-related TFs are also associated with ASD

Transcriptional targets of ASD-related TFs that were significantly dysregulated in the hippocampus of pups exposed to BPA in utero were analyzed by Ingenuity Pathway Analysis (IPA) software to predict biological functions, disorders, and pathways associated with these TF targets (Tables 2, 3, Additional files 7, 8, 9, 10, 11, 12, 13, 14). As AR and ESR1 are known BPA targets and are thought to be involved in the male bias of ASD [46, 48, 49], we, therefore, focused on these two TFs in this study. Interestingly, AR targets that were differentially expressed in male pups prenatally exposed to BPA were significantly associated with “autism spectrum disorder or intellectual disability” (*p* value = 1.04E−07) and “pervasive developmental disorder” (*p* value = 1.42E−04) (Table 2). Moreover, neurological functions known to be disrupted in ASD, including “proliferation of neuronal cells” (*p* value = 1.87E−05), “developmental process of synapse” (*p* value = 1.97E−04), and “excitatory postsynaptic potential” (*p* value = 8.25E−04) were also highlighted. ESR1 targets that were significantly altered in the hippocampus of male pups exposed to BPA were associated with “abnormal morphology of neurons” (*p* value = 3.22E−05), “neuritogenesis” (*p* value = 4.35E-05), and “proliferation of neuronal cells” (*p* value = 5.14E−05), all of which have been implicated in ASD (Table 3).

**Table 2:** Neurological diseases and functions associated with AR target genes that were differentially expressed in the hippocampus of male offspring prenatally exposed to BPA

Diseases or functions	*p *values	Genes	Number of genes
Neurological disorders			
Autism spectrum disorder or intellectual disability	1.04E−07	*Adnp*, *Chd2*, *Cic*, *Dst*, *Gria3*, *Kirrel3*, *Kmt2b*, *Kmt2c*, *Kmt2d*, *Mef2c*, *Nipbl*, *Nsd1*, *Slc25a12*, *Snap25*, *Sptan1*, *Syncrip*, *Tnrc6c*, *Wdfy3*, *Zic1*, *Zmynd8*	20
Microcephaly	1.16E−05	*Adnp*, *Hspg2*, *Kmt2c*, *Kmt2d*, *Nsd1*, *Sptan1*, *Tp53bp1*, *Trps1*, *Wdfy3*	9
Growth failure or short stature	3.08E−05	*Ckb*, *Eif2ak3*, *Hspg2*, *Itpkb*, *Jup*, *Kdm2a*, *Kmt2b*, *Kmt2c*, *Lrp1*, *Mef2c*, *Tial1*, *Tp53bp1*, *Trps1*, *Txnip, Zic1*	15
Abnormal morphology of embryonic tissue	9.06E−05	*Adnp*, *Epn1*, *H2az1*, *Itgb8*, *Jup*, *Kdm2a*, *Kmt2b*, *Mef2c*, *Nsd1*, *Pam*, *Pou3f3*, *Zfpm1*	12
Pervasive developmental disorder	1.42E−04	*Adnp*, *Dst*, *Kirrel3*, *Kmt2b*, *Mef2c*, *Slc25a12*, *Syncrip*, *Wdfy3*	8
Global developmental delay	1.75E−04	*Adnp*, *Elavl2*, *Gria3*, *Kmt2b*, *Nipbl*, *Pou3f3*, *Snap25*	7
Nervous system development and functions			
Proliferation of neuronal cells	1.87E−05	*Adnp*, *Dagla*, *Epha2*, *Epn1*, *Erbb4*, *Kdm2a*, *Map1b*, *Otp*, *Pkp4*, *Sema4c*, *Snap25*, *Spock2*, *Zfyve26*	13
Developmental process of synapse	1.97E−04	*Adnp*, *Caprin1*, *Epha7*, *Erbb4*, *Kirrel3*, *Mef2c*, *Slitrk1*	7
Excitatory postsynaptic potential	8.25E−04	*Caprin1*, *Gria3*, *Mef2c*, *Rims2*, *Snap25*	5
Docking of synaptic vesicles	1.36E−03	*Rims2*, *Snap25*	2
Neuritogenesis	1.55E−03	*Caprin1*, *Ckb*, *Csmd3*, *Epha7*, *Erbb4*, *Kirrel3*, *Lrp1*, *Map1b*, *Neo1*, *Pkp4*, *Slitrk1*	11
Behaviors			
Abnormal posture	5.75E−04	*Caprin1*, *Eif2ak3*, *Trps1*, *Zfyve26*	4
Cognition	3.13E−03	*Adnp*, *Cic*, *Ckb*, *Erbb4*, *Kmt2b*, *Kmt2d*, *Nipbl*, *Snap25*, *Zfpm1*	9

Biological functions and diseases associated with AR transcriptional targets that were differentially expressed in the hippocampus of male offspring prenatally exposed to BPA were predicted using IPA software. Statistical significance was determined using Fisher’s exact test. A *p* value < 0.05 was considered significant

**Table 3:** Neurological diseases and functions associated with ESR1 target genes that were differentially expressed in the hippocampus of male offspring prenatally exposed to BPA

Diseases or functions	*p* values	Genes	Number of genes
Neurological disorders			
Growth failure or short stature	1.58E−07	*Agps, Braf, Cdk13, Dchs1, Efnb2, Fgfr2, Fgfr3, Gja1, Ip6k1, Kdm2a, Kmt2e, Rev3l, Tial1, Trps1, Zbtb20*	15
Autosomal dominant mental retardation	1.37E−05	*Cdk13, Chd2, Cic, Kirrel3, Kmt2e, Zbtb20*	6
Saethre–Chotzen syndrome	2.07E−05	*Fgfr2, Fgfr3*	2
Dwarfism	4.29E−04	*Agps, Fgfr2, Fgfr3, Gja1, Trps1*	5
Familial congenital anomaly of limb	4.98E−04	*Fgfr2, Fgfr3, Gja1, Psd3, Trps1*	5
Nervous system development and functions			
Abnormal morphology of neurons	3.22E−05	*B4galnt1, Braf, Cpeb3, Epha7, Fgfr3, Gja1, Itgb8, Kif5a, Map1a, Wnt4*	10
Neuritogenesis	4.35E−05	*Arfgef1, Braf, Cpeb3, Efnb2, Epha7, Fgfr2, Fgfr3, Gja1, Kirrel3, Plxnb1, Rhoq*	11
Proliferation of neuronal cells	5.14E−05	*Arfgef1, B4galnt1, Braf, Efnb2, Fgfr2, Fgfr3, Gja1, Kdm2a, Rhoq, Spock2*	10
Branching morphogenesis of nerves	6.86E−05	*Epha7, Fgfr2*	2

Biological functions and diseases associated with ESR1 transcriptional targets that were differentially expressed in the hippocampus of male offspring prenatally exposed to BPA were predicted using IPA software. Statistical significance was determined using Fisher’s exact test. A *p* value < 0.05 was considered significant

Transcriptional target genes of EGR2, KDM5B, SOX5, SMAD4, TCF7L2, and YY1 that were differentially expressed in the BPA group were also analyzed by IPA (Additional files  7, 8, 9, 10, 11, 12, 13, 14). In addition to AR targets, KDM5B targets dysregulated in male and female pups, and SMAD4 targets dysregulated in male pups were also significantly associated with “autism spectrum disorder or intellectual disability” (Additional files 8, 9, 10). These results suggest that transcriptional targets of these ASD-related TFs that were differentially expressed in the offspring hippocampus are associated with ASD, and BPA may increase the susceptibility of ASD in male and female offspring by disrupting the expression of genes related to ASD through different TFs. AR, KDM5B, and SMAD4 may be involved in the effects of prenatal BPA exposure on the expression of ASD candidate genes in male offspring, whereas only KDM5B may be involved in females.

### Molecular docking analysis of BPA and ASD-related TFs whose targets were differentially expressed in the hippocampus of offspring prenatally exposed to BPA

**Fig. 1:**
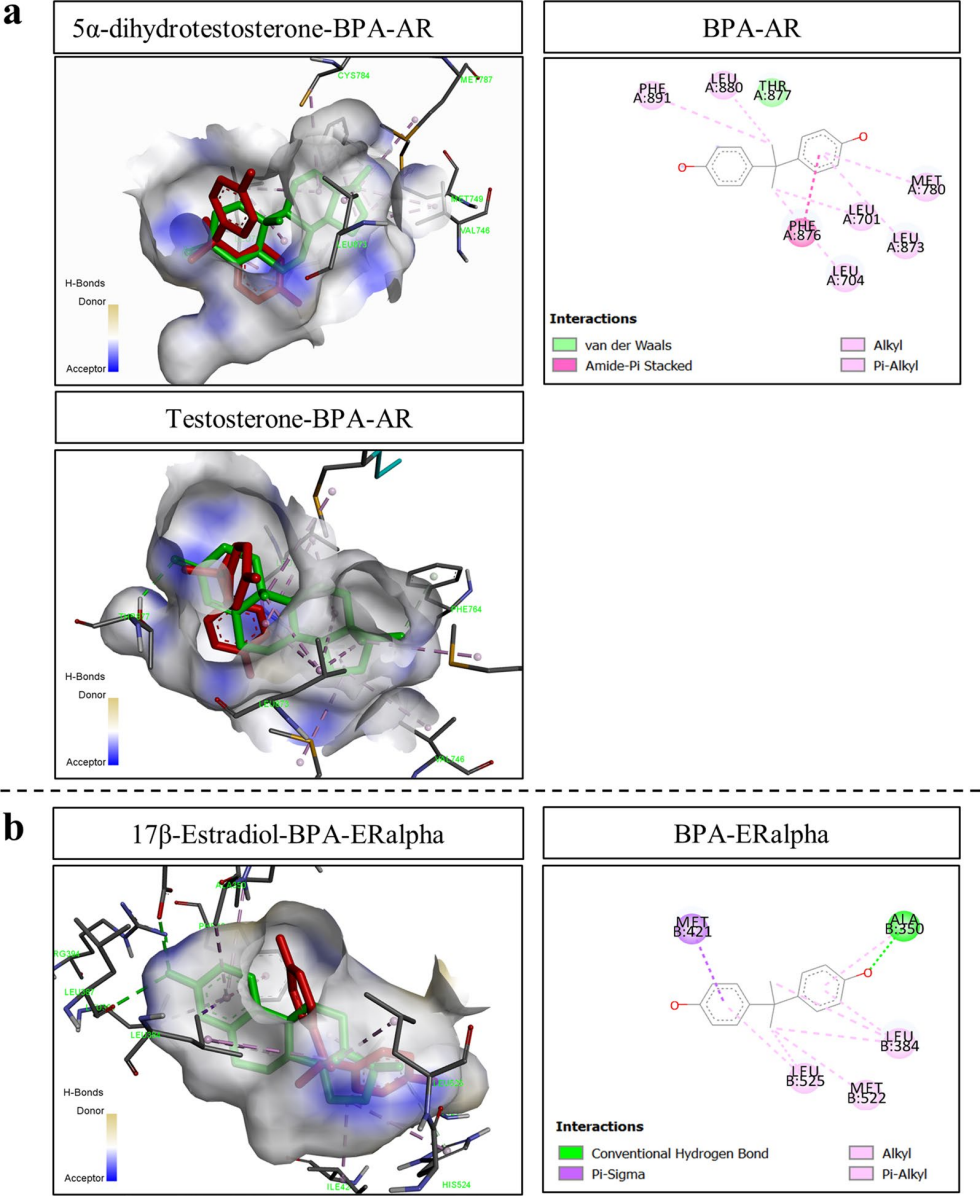
**Molecular docking analysis of BPA and AR or ESR1 molecules. **Direct interactions between BPA and androgen receptor (AR) (**a**) or estrogen receptor alpha (ESR1) (**b**) were analyzed using Discovery Studio 2020 and AutoDock 4.2 software. The 3D structures of BPA (red) and the ligands (green) were obtained from the NCBI PubChem database, and the structures of AR and ERalpha (ESR1) were obtained from the RCSB PDB Protein Data Bank database. The amino acids interacting with the BPA molecule and types of interactions were also demonstrated

**Fig. 2:**
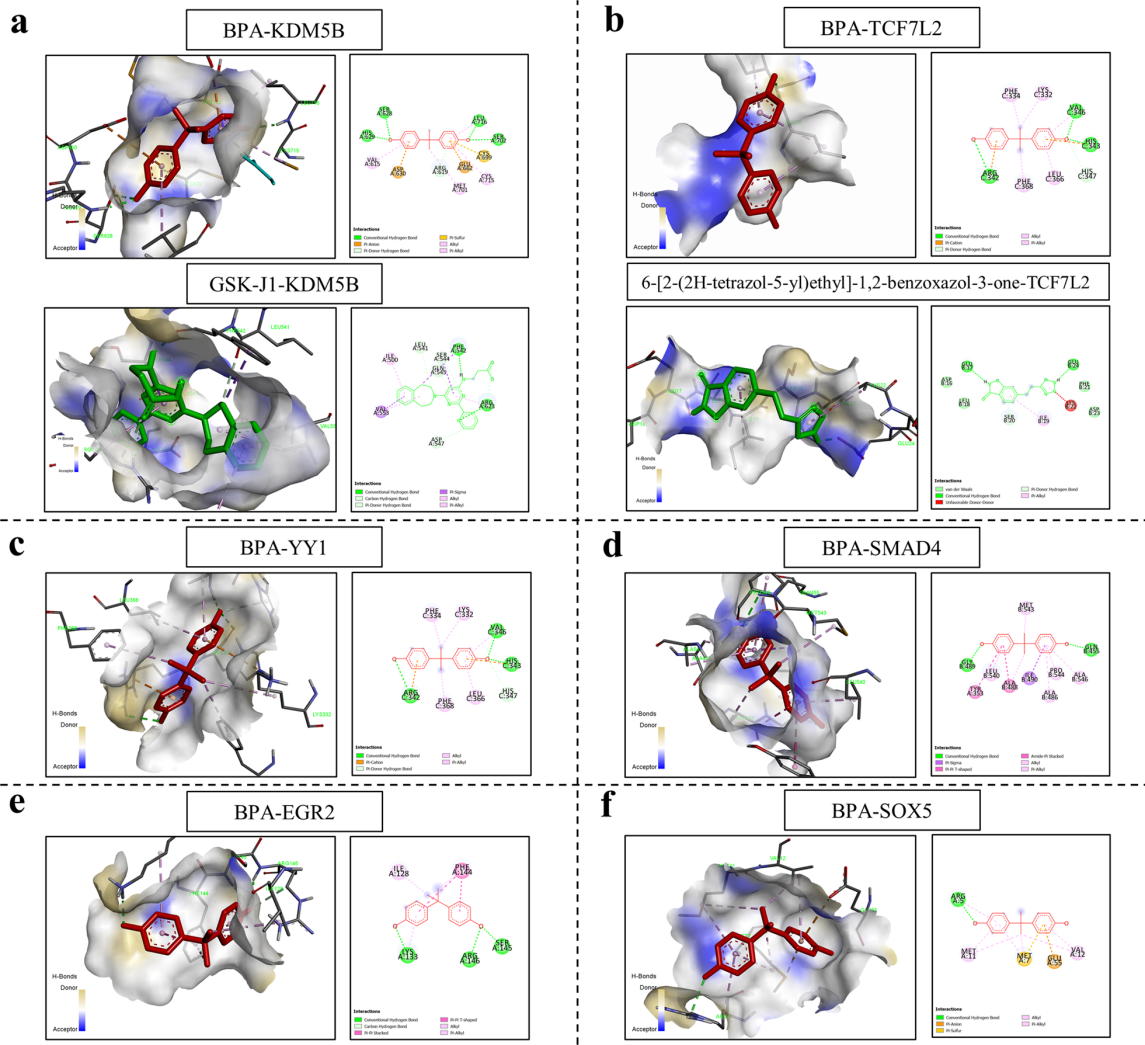
**Molecular docking analysis of BPA and other ASD-related TFs whose target genes were significantly associated with BPA-responsive genes in the hippocampus.** Molecular docking analysis of BPA and ASD-related TFs, KDM5B (**a**), TCF7L2 (**b**), YY1 (**c**), SMAD4 (**d**), EGR2 (**e**), and SOX5 (**f**), was performed using Autodock 4.2 software and Discovery Studio 2020. BPA is shown in red, and the ligands are shown in green

To examine whether BPA can directly interact with ASD-related TFs, the binding affinity between BPA and each ASD-related TF molecule was assessed using molecular docking analysis with Discovery Studio 2020 and AutoDock 4.2 software. Known ligands of each TF, when available, were also included for comparisons. Molecular docking analysis of BPA and the 8 ASD-related TFs whose targets were differentially expressed in response to prenatal BPA exposure showed that BPA directly interacted with AR, ESR1, EGR2, KDM5B, SOX5, SMAD4, TCF7L2, and YY1 (Table 4). Notably, SMAD4, KDM5B, and TCF7L2 have not been reported to interact with BPA, suggesting that these ASD-related TFs may serve as novel targets of BPA. When compared to known ligands, BPA interacted with AR at the same binding sites with 5α-dihydrotestosterone and testosterone and interacted with ESR1 at the same binding sites with 17β-estradiol (Fig. 1a, b). Unlike AR and ESR1, BPA interacted with KDM5B and TCF7L2 at different locations from their ligands, GSK-J1 and 6-[2-(2H-tetrazol-5-yl)ethyl]-1,2-benzoxazol-3-one, respectively (Fig. 2a, b). It is noteworthy that three TFs (i.e., AR, ESR1, and SMAD4) that interacted with BPA with low binding free energies also showed a male-specific enrichment of their transcriptional targets in the list of BPA-responsive genes (Table 1), suggesting that BPA may exert male-specific effects on the expression of ASD candidate genes in the offspring hippocampus and related functions through direct interactions with these TFs.

**Table 4:** Molecular docking analysis of BPA and ASD-related TFs

TFs (PDB ID) Refs.	Name	Known ligands					BPA			
		Name	Δgbind (kcal/mol)	Amino acid interaction			Δgbind (kcal/mol)	Amino acid interaction		
				Hydrogen bond	Hydrophobic bond	Electrostatic bond		Hydrogen bond	Hydrophobic bond	Electrostatic bond
SMAD4 (1DD1) Qin et al., 1999	Mothers against decapentaplegic homolog 4	NA	NA	NA	NA	NA	− 7.92 ± 0.12	GLN455, GLY489	TYR353, ALA486, ALA488, ILE490, LEU540, MET543, PRO544, ALA546	NA
KDM5B (5A3N) Tumber et al., 2017	Lysine Demethylase 5B	GSK-J1	− 7.81 ± 0.72	LEU541, PHE542, SER544, GLN545, ASP547, LEU621, ARG623	ILE500, PHE542, SER544, VAL553, ARG623	NA	− 7.60 ± 0.27	ARG619, SER628, HIS629, SER702, LEU716	VAL615, MET701, CYS715, LEU716	ASP630, GLU662, CYS699
AR (2Q7I) Askew et al., 2007	Androgen receptor	DHT	− 9.19 ± 0.04	PHE764	LEU704, LEU707, VAL746, MET749, PHE764, CYS784, MET787, LEU873	NA	− 7.02 ± 0.01	NA	LEU701, LEU704, MET780, LEU873, PHE876, LEU880, PHE891	NA
		Testosterone	− 9.18 ± 0.01	PHE764, THR877	LEU704, LEU707, MET742, VAL746, MET780, MET787, LEU873	NA				
ESR1 (1A52) Tanenbaum et al., 1998	Estrogen receptor alpha	17β-estradiol	− 8.78 ± 0.06	GLU353, ARG394, HIS524	LEU349, ALA350, LEU384, LEU391, PHE404, MET421, ILE424, HIS524, LEU525	NA	− 6.71 ± 0.06	ALA350	ALA350, LEU384, MET421, MET522, LEU525	NA
EGR2 (1A1I) Elrod-Erickson et al., 1998	Early growth response protein 2	NA	NA	NA	NA	NA	− 6.68 ± 0.04	SER145, ARG146, LYS133	ILE128, PHE144	NA
YY1 (1UBD) Houbaviy et al., 1996	Yin Yang 1 transcriptional repressor protein	NA	NA	NA	NA	NA	− 5.48 ± 0.03	HIS343, VAL346, ARG342, HIS347	LYS332, PHE334, VAL346, LEU366, PHE368	ARG342, HIS343
SOX5 (1I11) Cary et al., 2008	SRY-box 5	NA	NA	NA	NA	NA	− 5.44 ± 0.03	ARG5	ARG5, MET7, MET11, VAL12	MET7, GLU55
TCF7L2 (1JDH) Graham et al., 2001	Transcription factor 7-like 2 (T-cell specific, HMG-box)	6-[2-(2H-tetrazol-5-yl)ethyl]-1,2-benzoxazol-3-one	− 5.13 ± 0.41	GLU17, SER20, GLU24	ILE19	LYS22	− 4.33 ± 0.02	ILE19	LEU18, ILE19	NA

Molecular docking analysis of BPA and ASD-related TFs whose target genes were differentially expressed in the hippocampus of rat pups prenatally exposed to BPA was conducted using Discovery Studio 2020 and AutoDock 4.2 software. The 3D structures of BPA and ligands were obtained from the NCBI PubChem database, and the structures of the TFs were obtained from the RCSB PDB Protein Data Bank database. Average binding free energies and standard deviations were calculated from three runs

*NA* not available

### Effects of prenatal BPA exposure on the expression of ASD-related TFs

**Fig. 3:**
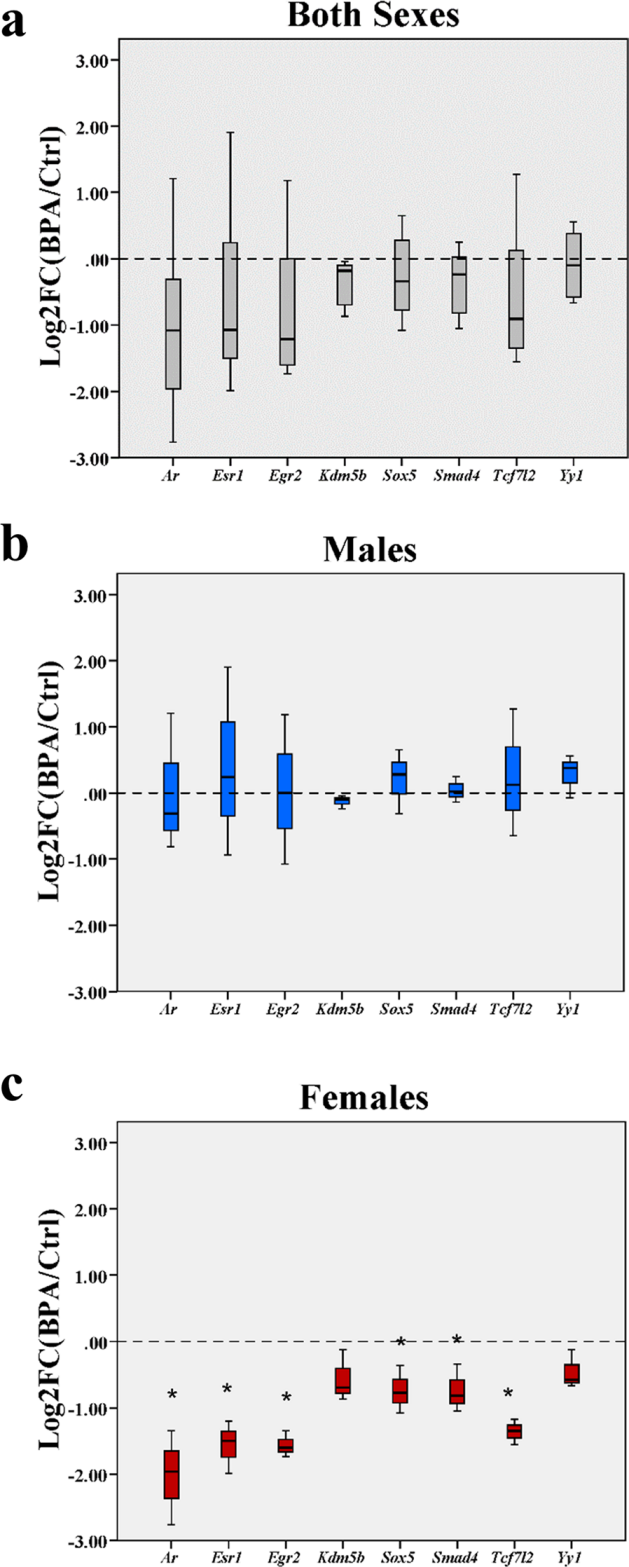
**Quantitative RT-PCR analysis of ASD-related TFs in the hippocampus of offspring prenatally exposed to BPA or vehicle control.** The expression levels of eight ASD-related TFs (i.e., *Ar*, *Esr1*, *Egr2*, *Kdm5b*, *Sox5*, *Smad4*, *Tcf7l2*, and *Yy1*) whose targets were differentially expressed in response to BPA were analyzed by qRT-PCR analysis using the hippocampus of pups prenatally exposed to BPA (*n* = 6 pups; 3 males and 3 females from 3 different dams) or vehicle control (*n* = 6 pups; 3 males and 3 females from 3 different dams). Data are presented as the mean ± SEM. The differences between the two groups were analyzed using the two-tailed Student’s *t* test. A *p*-value < 0.05 was considered significant. *p value < 0.05

In addition to direct interactions with TFs, BPA may disrupt the expression of ASD candidate genes in the offspring hippocampus through other pathways that affect the expression of TFs [32, 34, 77]. To examine the effects of prenatal BPA exposure on the expression of ASD-related TFs in the hippocampus of offspring, we first assessed whether these ASD-related TFs were expressed in the developing brain of rodents by obtaining the expression data of these TFs in embryonic and early postnatal stages of rodents from the Allen Brain Atlas database (Additional file 15). Seven of these TFs (i.e., *Ar*, *Esr1*, *Egr2*, *Kdm5b*, *Sox5*, *Smad4*, and *Tcf7l2*) were expressed in the rostral secondary prosencephalon and the telencephalic vesicle of the developing brain (Additional file 15). The expression data of *Yy1* were not available. Moreover, qRT-PCR analysis of hippocampal tissues isolated from rat pups (PND1) prenatally exposed to BPA (*n* = 6 pups; 3 males and 3 females from 3 different dams) or vehicle control (*n* = 6 pups; 3 males and 3 females from 3 different dams) was conducted (Fig. 3). When male and female pups in the same treatment group were combined into one group, the expression levels of ASD-related TFs were not significantly changed. However, when each sex of pups was analyzed separately, the qRT-PCR analysis revealed a sex difference in the effects of prenatal BPA exposure on the expression of these TFs. In male pups, the expression of these TFs in the hippocampus was not impacted by prenatal BPA exposure, but in females, the expression levels of several TFs (i.e., *Ar*, *Esr1*, *Egr2*, *Sox5*, *Smad4*, and *Tcf7l2*) were significantly reduced (Fig. 3). This result suggests that, unlike in males, BPA may also alter the transcriptome profiles in the hippocampus of female offspring by suppressing the expression of these ASD-related TFs in addition to direct interactions with TFs.

### Quantitative RT-PCR analysis of transcriptional targets of ASD-related TFs in the hippocampus of male and female offspring

**Fig. 4:**
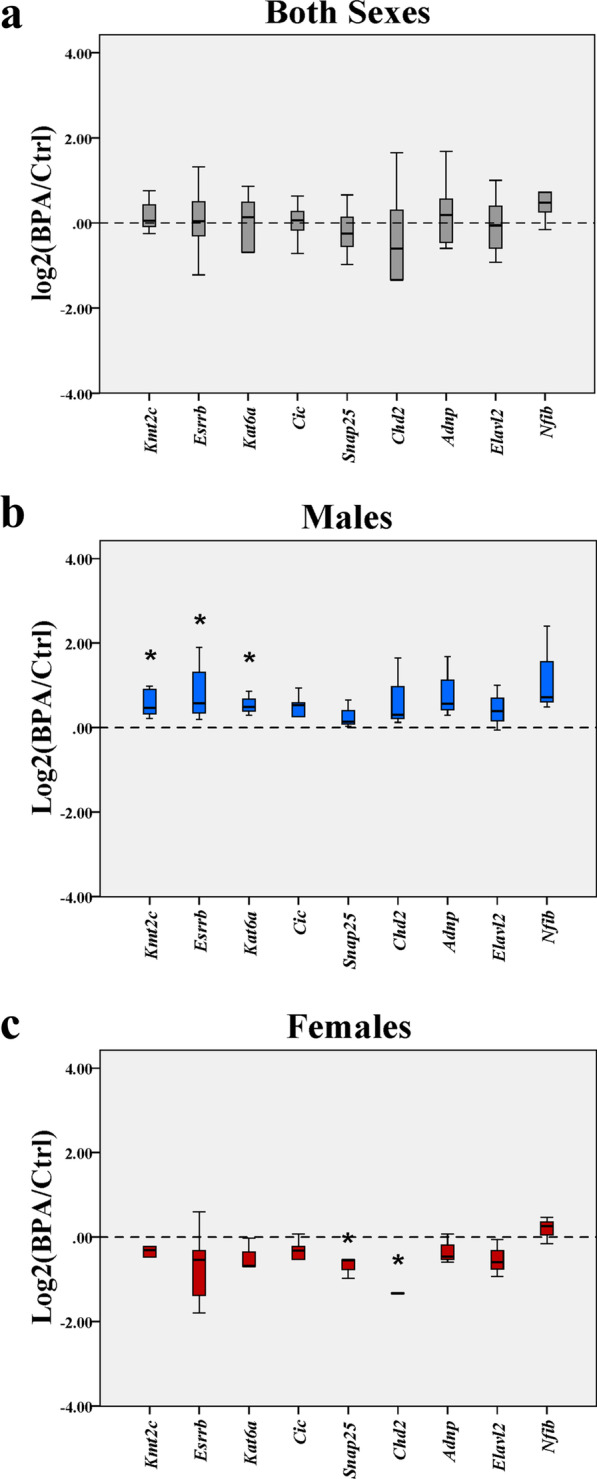
**Quantitative RT-PCR analysis of transcriptional targets of ASD-related TFs in the hippocampus of offspring prenatally exposed to BPA or vehicle control.** Quantitative RT-PCR analysis was performed to determine the expression levels of selected transcriptional targets of ASD-related TFs using the hippocampus of pups prenatally exposed to BPA (*n* = 6 pups; 3 males and 3 females from 3 different dams) or vehicle control (*n* = 6 pups; 3 males and 3 females from 3 different dams). Data are presented as the mean ± SEM. The differences between the two groups were analyzed using the two-tailed Student’s *t*-test. A *p* value < 0.05 was considered significant. *p value < 0.05

To confirm the sex difference in the expression of genes that were transcriptional targets of ASD-related TFs and were dysregulated in the hippocampus of offspring, qRT-PCR analysis was performed using hippocampal tissues from rat pups (PND1) prenatally exposed to BPA (*n* = 6 pups; 3 males and 3 females from 3 different dams) or vehicle control (*n* = 6 pups; 3 males and 3 females from 3 different dams). A total of nine genes (i.e., *Adnp, Chd2, Cic, Elavl2, Esrrb, Kat6a, Kmt2c*, *Nfib,* and *Snap25*) were selected for qRT-PCR analysis. These genes have been identified by the TRANSFAC Curated database to be transcriptional targets of AR, ESR1, EGR2, SOX5, and YY1, which showed a binding free energy of less than − 5 kcal/mol when interacting with BPA (Table 4). Moreover, the expression of *Ar*, *Esr1*, *Egr2*, and *Sox5* was downregulated in the hippocampus of female offspring in response to prenatal BPA exposure but not in males (Fig. 3). Information about each ASD-related TF and its transcriptional targets that were selected for qRT-PCR analysis in this study is provided in Additional file 16. When both sexes of pups in the same treatment group were combined into one group, we found that there was no significant difference in the expression of these TF target genes in the BPA group (Fig. 4a). However, when each sex of pups was analyzed separately, we found a sex difference in the effects of prenatal BPA exposure on the expression of these genes. The expression of *Kmt2c*, *Esrrb*, and *Kat6a* was significantly upregulated in males but not in females (Fig. 4b, c), whereas the expression of *Snap25* and *Chd2* was significantly decreased in females only (Fig. 4c). Notably, *Kmt2c*, *Esrrb*, *Snap25*, and *Chd2* are transcriptional targets of AR. *Kat6a* is a transcriptional target of SOX5. In addition to these nine genes, we conducted a qRT-PCR analysis of the hippocampus of rat offspring prenatally exposed to BPA and vehicle control in our previous study and found that *Auts2*, *Dicer1*, *Foxp2*, and *Smarcc2* were differentially expressed in response to BPA [13]. These genes are also transcriptional targets of the ASD-related TFs and, therefore, included in the subsequent analysis (Additional file 16). Notably, *Auts2* and *Foxp2* are transcriptional targets of AR.

**Fig. 5:**
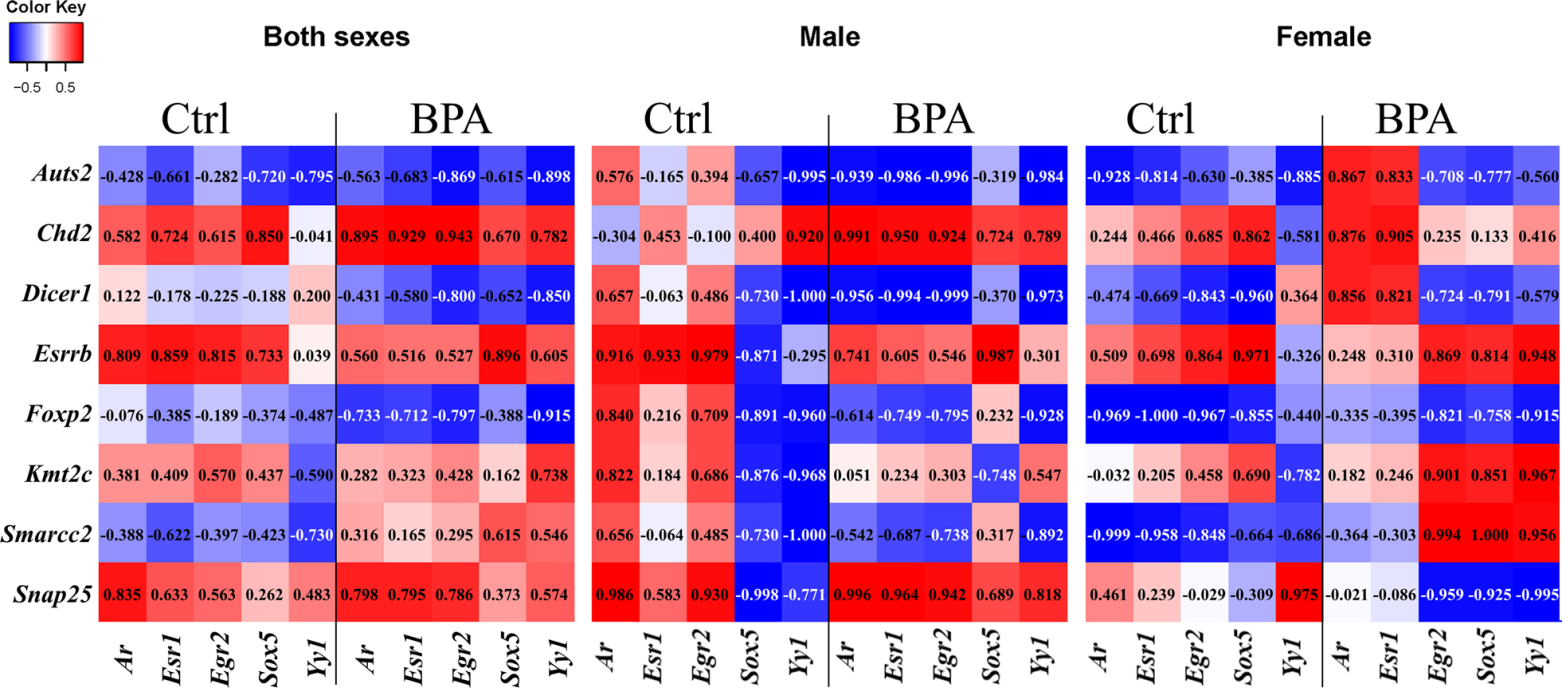
**Heatmap of the correlation matrix between the expression levels of TFs and target genes in the hippocampus of offspring.** The correlations between the expression levels of TFs (i.e., *Ar*, *Esr1*, *Egr2*, *Sox5*, and *Yy1*) and target genes (i.e., *Auts2*, *Chd2*, *Dicer1*, *Esrrb*, *Foxp2*, *Kmt2c*, *Smarcc2*, and *Snap25*) in the hippocampus of rat pups prenatally exposed to BPA or the vehicle control were determined using Pearson’s correlation. The color scale denotes *R*^2^ values from red (positive correlation) to blue (negative correlation)

To examine the relationships between the expression of ASD-related TFs and transcription targets that were differentially expressed in the offspring hippocampus, a correlation analysis was performed using the expression levels of ASD-related TFs (i.e., *Ar*, *Esr1*, *Egr2*, *Sox5*, and *Yy1*) and their transcriptional targets (i.e., *Auts2, Chd2, Dicer1, Esrrb, Foxp2, Kmt2c, Smarcc2*, and *Snap25*) from the qRT-PCR analysis (Fig. 5). Interestingly, the correlation analysis revealed sex-dependent relationships between these TFs and the transcriptional targets. The expression levels of AR and its targets (i.e., *Auts2, Chd2, Esrrb, Foxp2, Kmt2c,* and *Snap25*) were strongly correlated in a sex-specific pattern in response to prenatal BPA exposure, suggesting that AR is involved in the sex-specific effects of BPA on the expression levels of these ASD candidate genes.

### AR is involved in the effects of BPA on the expression of ASD-candidate genes in the human neuroblastoma cell line

**Fig. 6:**
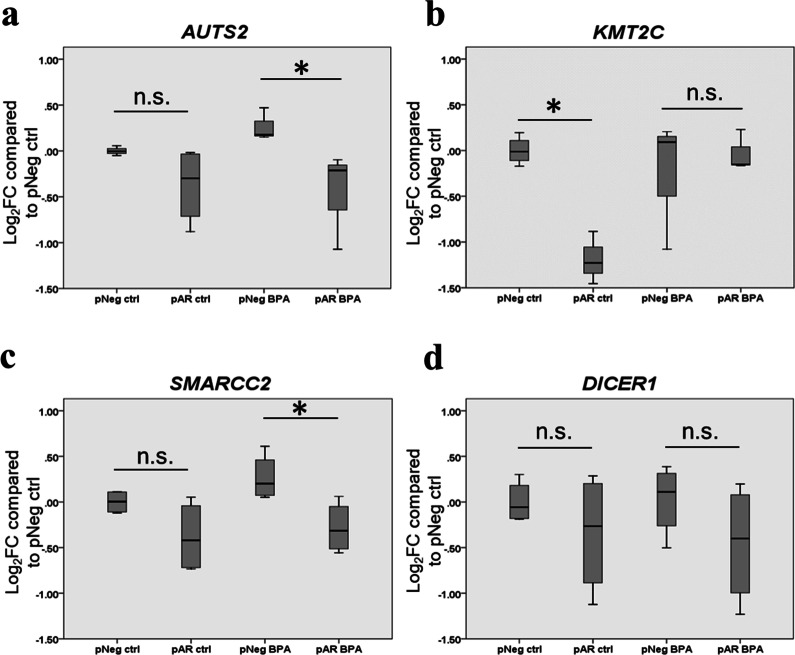
**AR is involved in the effects of BPA on the expression of** ***AUTS2*,** ***KMT2C*,** **and** ***SMARCC2*** **in the human neuroblastoma cell line SH-SY5Y.** The cell line stably transfected with AR-overexpression plasmid (pAR) or negative control plasmid (pNeg) was treated with BPA (*n* = 5) or ethanol as a vehicle control for treatment (*n* = 5). The expression levels of representative genes transcriptionally regulated by AR and other ASD-related TFs were assessed using qRT-PCR analysis. Data are presented as the mean ± SEM. The differences between the two groups were analyzed using the two-tailed Student’s *t* test. A *p* value < 0.05 was considered significant. *p value < 0.05

To further determine whether AR is also involved in the effects of BPA on the expression of ASD-candidate genes in human neuronal cells, the human neuroblastoma cell line SH-SY5Y stably transfected with AR-expression plasmid (pAR) or negative control plasmid (pNeg) was used. The expression levels of AR in the transfected cells were assessed using Western blot analysis (Additional file 4). The SH-SY5Y cells stably transfected with the negative control plasmid exhibited very low expression of AR proteins, while the cells transfected with the AR-expression plasmid showed a significant increase in AR protein expression. We also examined the expression of estrogen receptors in the transfected cells and found that the levels of *ESR1* and *ESR2* were not significantly changed (Additional file 17). These cells were differentiated into mature neurons and were treated with 1 ng/mL BPA (*n* = 5) or the vehicle control (*n* = 5) during differentiation. The concentration of BPA used for treatment in this experiment was calculated from an estimated BPA level in the fetus's brain when 5000 µg/kg·maternal body weight of BPA was given to a pregnant rat [78]. After 7 days of differentiation, RNA was extracted from the cells, and qRT-PCR analysis of selected ASD candidate genes (i.e., *AUTS2*, *ESRRB*, *KMT2C*, *SMARCC2*, and *DICER1*) was performed (Fig. 6). *ESRRB* expression in the SH-SY5Y cells transfected with pAR or pNeg plasmid was undetectable by qRT-PCR analysis and thus excluded from data analysis.

The expression patterns of *AUTS2*, *KMT2C*, and *SMARCC2* in the cells with AR overexpression (pAR) and the cells with low AR levels (pNeg) were changed in response to BPA treatment (Fig. 6). Without BPA, the expression levels of *AUTS2* and *SMARCC2* tended to decrease but not significantly when AR was overexpressed (Fig. 6a, c). However, in the presence of BPA, the expression levels of these genes in the cells with AR overexpression were significantly reduced when compared to the cells with low AR expression levels. In contrast, *KMT2C* expression in cells stably transfected with the pAR plasmid was lower than that in cells transfected with the negative control plasmid (Fig. 6b). When treated with BPA, the expression of *KMT2C* in the cells with AR overexpression was significantly increased compared to cells treated with the vehicle control and exhibited no significant difference when compared to cells with low AR expression treated with BPA. These results suggest that AR, together with other mechanisms, regulates the expression of these ASD candidate genes, and BPA suppresses *AUTS2* and *SMARCC2* expression and enhances *KMT2C* expression through AR.

### Sex differences in the effects of prenatal BPA exposure on synaptogenesis

**Fig. 7:**
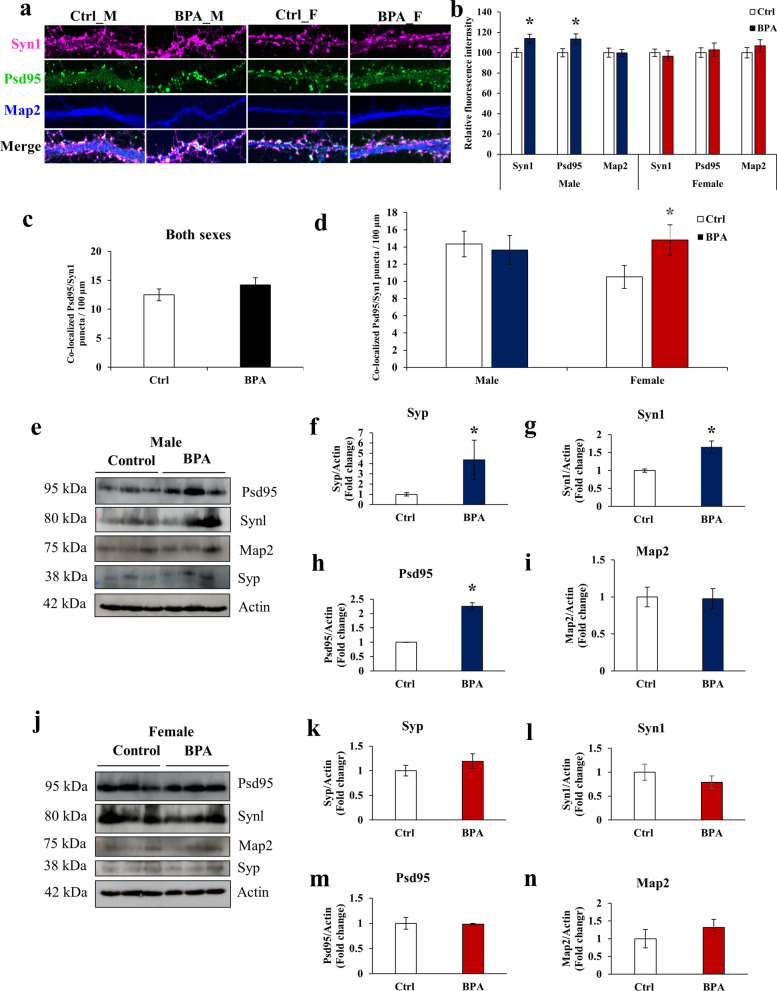
**Prenatal BPA exposure disrupts synaptogenesis in the hippocampus of offspring in a sex-dependent manner.** **a**–**d** Primary hippocampal neurons were isolated from neonatal rat pups (PND1) prenatally exposed to BPA or vehicle control. The hippocampal tissues from each sex of pups in the same litter were pooled into one sample and used for cell isolation. For each treatment group, a total of three samples for each sex of pups were used. Primary hippocampal neurons were cultured for 14 days and immunostained with rabbit anti-Syn1 (presynaptic marker), mouse anti-Psd95 (postsynaptic marker), and chicken anti-Map2 antibodies (mature neuron marker), followed by donkey anti-rabbit Alexa 647, donkey anti-mouse Alexa 488, and donkey anti-chicken Alexa 405 antibodies. The cells were then examined under a confocal laser scanning microscope. For each treatment group, a total of 45 mature neurons/sex were assessed. For each neuron, all neurites (approximately 3–5 neurites/neuron) were used for data analysis. **a** Representative immunofluorescence images of synapses on primary hippocampal neurons isolated from rat pups prenatally exposed to BPA or vehicle control. **b** Relative fluorescence intensities of Syn1, Psd95, and Map2 in primary hippocampal neurons (DIV14) from male and female pups exposed to BPA or vehicle control in utero. **c** Average number of colocalized Syn1 and Psd95 puncta per 100 µm when both sexes of pups in the same treatment group were combined into one group. **d** Average number of colocalized Syn1 and Psd95 puncta per 100 µm when male and female pups were analyzed separately. **e**–**n** Expression levels of synaptic proteins in the hippocampus of rat pups (*n* = 6 pups/treatment group; 3 males and 3 females from 3 different dams) were also assessed by Western blot analysis. **e**–**i** Expression levels of Syp, Syn1, Psd95, and Map2 proteins in the hippocampal tissues of male offspring prenatally exposed to BPA or control. **j**–**n** Expression levels of Syp, Syn1, Psd95, and Map2 proteins in the hippocampal tissues of female offspring prenatally exposed to BPA or control. Data are presented as the mean ± SEM. The differences between the two groups were analyzed using the two-tailed Student’s *t*-test. A *p *value < 0.05 was considered significant. *p value < 0.05

Several AR targets (i.e., *Adnp*, *Auts2*, *Snap25*) that were selected for confirmation by qRT-PCR analysis and differentially expressed in the BPA group are known to play an important role in synaptogenesis and synaptic functions associated with ASD [79–81]. For instance, the reduction of the AUTS2 level leads to increased excitatory synapses in the brain [80]. To investigate the effects of prenatal BPA exposure on synaptogenesis, we isolated primary hippocampal neurons at PND1 from neonatal rat pups prenatally exposed to BPA or vehicle control. Primary hippocampal neurons were cultured for 14 days and immunostained for the neuronal marker protein Map2, the presynaptic protein Syn1, and the postsynaptic protein Psd95 (Fig. 7a). The fluorescence intensity of each marker and the colocalization of the presynaptic and postsynaptic markers were examined. We found that the expression levels of the presynaptic marker Syn1 and the postsynaptic marker Psd95 were significantly increased in primary hippocampal neurons from males prenatally exposed to BPA but not in those from females (Fig. 7b). Conversely, the number of colocalized Syn1 and Psd95 foci was significantly increased only in female primary hippocampal neurons (Fig. 7d). To further examine the expression of synaptic proteins in hippocampal tissues, Western blot analysis of hippocampal tissues from rat pups prenatally exposed to BPA (*n* = 6 pups; 3 males and 3 females from 3 different dams) or vehicle control (*n* = 6 pups; 3 males and 3 females from 3 different dams) was performed (Fig. 7e–n). When both sexes of pups in the same treatment group were combined, no changes in the expression of Map2 and these synaptic proteins were observed (data not shown). However, when each sex of pups was analyzed separately, the Western blot analysis showed significant upregulation of Syp, Syn1, and Psd95 proteins only in male hippocampal tissues (Fig. 7e–h) but not in female hippocampal tissues (Fig. 7j–m). No significant change in the expression of Map2 was observed in either sex (Fig. 7i, n). These results indicate that prenatal exposure to BPA causes sex-specific effects on the expression of synaptic proteins and synaptogenesis.

### Reanalysis of transcriptome data from other BPA studies revealed dysregulation of ASD-related TF targets in the hippocampus of offspring

To determine whether ASD-related TFs might be involved in the effects of prenatal BPA exposure on transcriptome profiles in the offspring hippocampus in independent studies, we obtained transcriptome data from other BPA studies conducted by independent groups of researchers from the NCBI PubMed database and reanalyzed them as described in the Methods. The details of each study, including the title, sample size, and treatment, are described in Additional file 18. The lists of DEGs in the offspring hippocampus in response to prenatal/perinatal BPA exposure from other BPA studies are shown in Additional file 19. Hypergeometric distribution analysis was performed to assess the enrichment of transcriptional targets of ASD-related TFs in the lists of BPA-responsive genes in the offspring hippocampus from each transcriptomic study (Additional file 20). Transcriptional targets of AR, ESR1, EGR2, KDM5B, SOX5, SMAD4, TCF7L2, and YY1 were also found to be differentially expressed in the hippocampus of rat/mouse pups prenatally/perinatally exposed to BPA in independent studies, supporting that these TFs may be involved in the effects of BPA on gene expression. It is noteworthy that transcriptional targets of AR, ESR1, SMAD4, and YY1 were also found to be significantly enriched in the list of BPA-responsive genes in the hippocampus of male pups reported by Zhang et al. [82].

## Discussion

The prevalence of ASD in males is approximately four times higher than that in females [17]. The exact cause and the mechanisms underlying the male bias of ASD are still unclear, but there is evidence that sex chromosomes and hormones are involved in ASD susceptibility. In the developing brain, sex hormones play an essential role in sex differences in brain structures, behaviors, cognition, and neurological functions associated with ASD [46–49]. Elevated concentrations of sex hormones, including testosterone, androstenedione, progesterone, and 17α-hydroxy-progesterone, were found in amniotic fluid samples of males who were later diagnosed with ASD [83]. The expression levels of estrogen receptors, aromatase (an enzyme that converts androgens to estrogen), and several estrogen receptor coactivators were also reduced in the frontal cortex of ASD individuals [40]. Moreover, our previous studies have shown that the expression of retinoic acid-related orphan receptor-alpha (RORA) was reduced in blood-derived cell lines and postmortem brain tissues from ASD individuals [84, 85]. This protein is differentially regulated by male and female sex hormones through AR and ESR1 and serves as an upstream regulator of several ASD-related genes, including *CYP19A1*, a gene encoding the aromatase enzyme that converts androgens to estrogen [25, 46, 48, 49]. Androgen negatively regulates the expression of RORA, whereas estrogen enhances the levels of RORA [46]. The findings from these studies serve as supporting evidence that sex hormones and their signaling are associated with ASD.

**Fig. 8:**
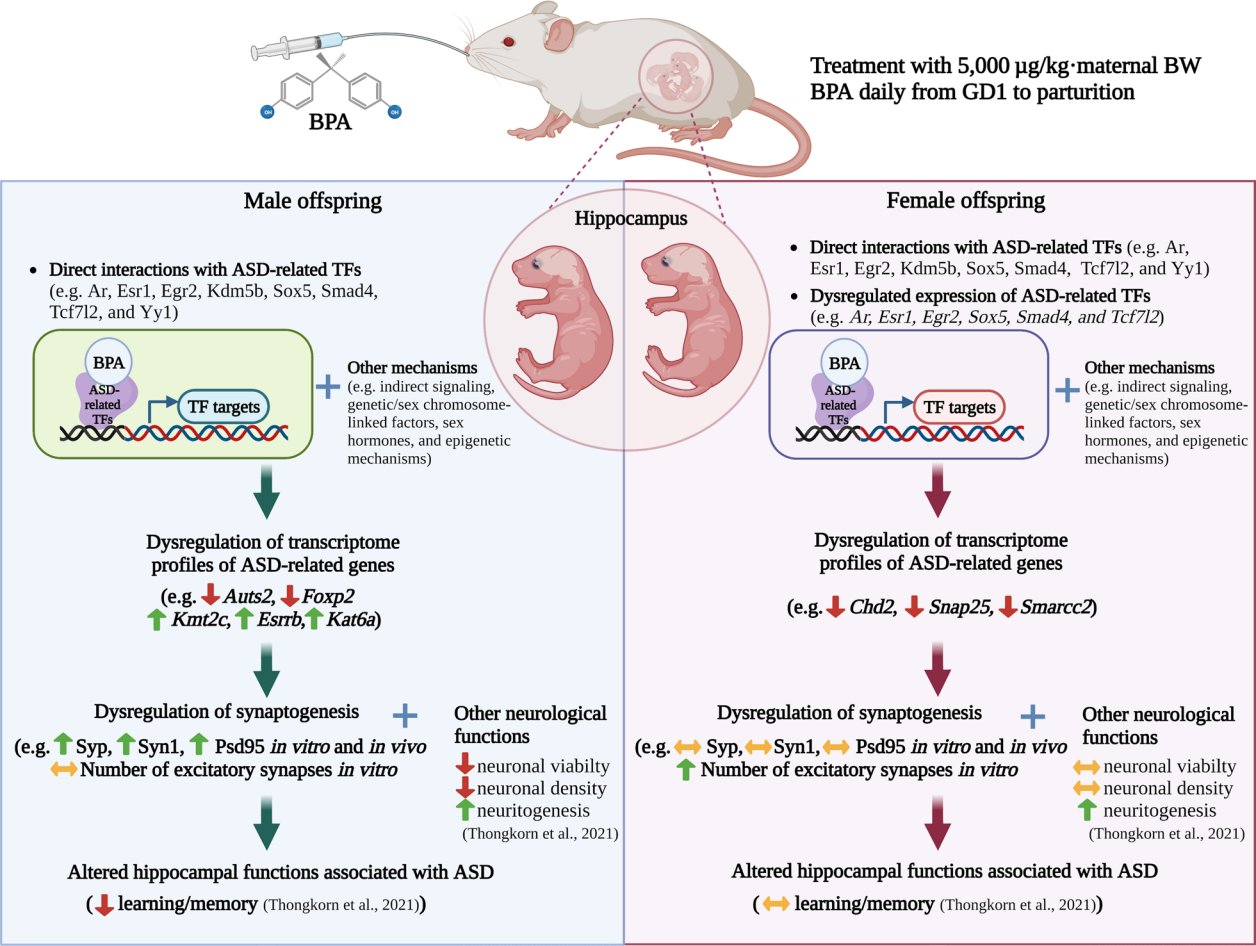
**Schematic diagram illustrating possible mechanisms underlying the effects of prenatal BPA exposure on the hippocampus of male and female offspring.** This figure was created with BioRender.com (http://biorender.com)

Both genetic and environmental factors can lead to abnormalities in sex hormones and their molecular signaling cascades during pregnancy, which may cause or increase the susceptibility of ASD in children [10, 13, 14, 18]. There is accumulating evidence that exposure to endocrine-disrupting chemicals, including BPA, phthalates, polychlorinated biphenyls (PCBs), and polybrominated diphenyl ethers (PBDEs), is an environmental risk factor for ASD [18, 86–88]. Our recent studies in rats have shown that maternal exposure to BPA during the gestation period altered the transcriptome profiles of genes associated with ASD and related neurological functions in the hippocampus and the frontal cortex of offspring in a sex-dependent manner [10, 13, 14]. Several ASD candidate genes, including Autism Susceptibility Gene 2 (*Auts2*) and Forkhead Box Protein P2 (*Foxp2*), were found to be significantly reduced in the hippocampus of male offspring but not in females in response to prenatal BPA exposure [13]. Moreover, we found that in utero BPA exposure increased neuritogenesis and reduced the size of the hippocampal cell body in both sexes of the offspring but decreased neuronal viability, the density of neuronal cells in the hippocampus, and learning/memory only in the male offspring [10]. Similarly, prenatal BPA exposure disrupted the transcriptome-interactome profiles of ASD candidate genes, including *Auts2*, *Ankrd11*, and *Ntng1*, in the offspring frontal cortex in a sex-specific pattern, possibly through AR, ESR1, and RORA [14]. These findings strongly suggest that BPA alters the expression of ASD-related genes and neurological functions in the offspring brain through sex-dependent mechanisms. However, the molecular mechanisms underlying the sex-specific effects of BPA have not been investigated. This is the first study to identify ASD-related TFs potentially involved in the sex differences in the effects of prenatal BPA exposure on the transcriptome profiles and neurological functions associated with ASD in the hippocampus of offspring. The major findings of our study are summarized in Fig. 8.

In this study, the rat dams were treated with BPA at 5000 µg/kg·maternal body weight or vehicle control from gestational day 1 until parturition by oral gavage. This dose of BPA is equal to the no-observed-adverse-effect level (NOAEL) for BPA exposure determined by the United States Food and Drug Administration (FDA) and the European Food Safety Authority (EFSA) [70]. Moreover, this dose was also used in our previous studies on the effects of prenatal BPA exposure on the transcriptome profiles of the hippocampus and hippocampal functions [10, 13], allowing for integrated interpretations of the results. The transcriptome profiling data from an RNA-seq analysis of hippocampal tissues isolated from male and female rat pups prenatally exposed to BPA or vehicle control were obtained from our previous study [13]. To determine whether BPA-responsive genes in the offspring hippocampus can be transcriptionally regulated by ASD-related TFs, we identified ASD-related TFs by overlapping the list of human TFs from the Human Transcription Factors database with the list of ASD candidate genes from the SFARI database (Additional file 5). In this study, we focused on ASD-related TFs, because there is evidence that several of them, including AR and ESR1, play an important role in the male bias of ASD [46–49] and thus serve as promising candidates for an investigation of the molecular mechanisms underpinning sex-specific effects of prenatal BPA exposure in the context of ASD. However, it is possible that, in addition to ASD-related TFs, BPA can also exert its effects through other TFs that have not been associated with ASD, which could be further investigated in the future.

Hypergeometric distribution analysis revealed that the transcriptional targets of AR, ESR1, KDM5B, SOX5, SMAD4, and YY1 were significantly enriched in the list of BPA-responsive genes in the hippocampus of male offspring. In contrast, the targets of EGR2, KDM5B, and SOX5 were significantly enriched in the list of BPA-responsive genes in females (Table 1). This result suggests that BPA may exert its effects on the transcriptome profiles in the hippocampus of male and female offspring through different TFs depending on the sex of the offspring. Although these TFs were obtained from a human transcription factor database, they are known to be highly conserved in mammals [89]. According to the Allen Brain Atlas database, all of these TFs, except Yy1, were also expressed in the developing brain of rodents during the prenatal stage (Additional file 15). Although there were no expression data for Yy1 in the database, our qRT-PCR analysis and results from other studies have confirmed that it is expressed in the developing brain (Fig. 3) [90, 91]. Moreover, molecular docking analysis showed that BPA can directly interact with these ASD-related TFs (Table 4). BPA is known to be an AR antagonist and an ESR1 agonist [92–94] and has been predicted to bind EGR2, SOX5, and YY1 directly [14]. However, direct interactions between BPA and KDM5B, SMAD4, or TCF7L2 have not been reported elsewhere, suggesting that these TFs are novel targets of BPA.

Gene ontology analysis by IPA software revealed that transcriptional targets of AR, KDM5B, and SMAD4 that were differentially expressed in response to BPA treatment were significantly associated with “autism spectrum disorder or intellectual disability” (Table 2, Additional files 8, 9, 10). Interestingly, the transcriptional targets of AR and SMAD4 were significantly enriched in the lists of BPA-responsive genes only in male offspring but not females, suggesting that BPA may exert its male-specific effects on neurological functions associated with ASD and increase the risk of ASD in male offspring through AR and SMAD4, which deserves further investigation. Moreover, the transcriptional targets of AR that were differentially expressed in the BPA group are significantly associated with “proliferation of neuronal cells,” “developmental process of synapse,” and “excitatory postsynaptic potential,” all of which have been linked to ASD [95–97]. Examples of AR targets associated with these neurological functions include *Adnp*, *Chd2*, *Cic*, *Kmt2c*, and *Snap25*. Interestingly, these genes are also ASD candidate genes in the SFARI database [98–102]. In addition, genetic abnormalities of these ASD-related TFs have been found in ASD individuals [44, 103–107]. Moreover, YY1 interacts with the methyl-CpG binding protein 2 (MECP2) protein and regulates the expression of *ANT1* encoding a mitochondrial adenine nucleotide translocase enzyme associated with Rett syndrome which is an ASD-related neurodevelopmental disorder with loss of motor skills and speech [108]. Taken together, these findings suggest that altered functions of these TFs, either caused by genetic mutations or BPA exposure, may lead to dysregulations of ASD-related genes and altered neurological functions, which, in turn, cause or increase ASD susceptibility. The effects of BPA binding on the activities and functions of these TFs should be further investigated.

In addition to direct interactions between BPA and these ASD-related TFs, we also investigated the effects of prenatal BPA exposure on the expression of genes encoding these ASD-related TFs in the hippocampus of offspring (Fig. 3). The qRT-PCR analysis revealed a sex difference in the effects of prenatal BPA exposure on the expression levels of *Ar*, *Esr1*, *Egr2*, *Sox5*, *Smad4*, and *Tcf7l2*. These genes were significantly suppressed in the hippocampus of female offspring prenatally exposed to BPA but not in males. It is noteworthy that BPA exposure has been reported to reduce the expression of *Esr1* in the hippocampus of offspring [109]. Chang et al. found that Sprague–Dawley rat pups exposed to BPA throughout gestation and lactation exhibited decreased *Esr1* expression in the hippocampus, along with elevated DNA methylation of the *Esr1* gene promoter [109]. In addition, Arambula et al. found that gestational exposure to BPA at 25,000 µg/kg maternal body weight tended to reduce the expression of *Esr1* in the hippocampus of female offspring but not in males, albeit not statistically significant [32]. These findings suggest that, in addition to direct interaction with these TFs, BPA also alters the expression of these TFs through other transcriptional regulatory mechanisms (e.g., DNA methylation), which are sex dependent. However, such effects may also depend on the dose and time of BPA exposure.

We further investigated the expression of transcriptional targets of these TFs in the offspring hippocampus by qRT-PCR analysis and found that prenatal BPA exposure altered the expression levels of AR target genes in a sex-dependent manner (Fig. 4). The expression levels of *Kmt2c* and *Esrrb* were increased in males but not in females, whereas the expression levels of *Snap25* and *Chd2* were reduced in females only. Moreover, our previous study found that *Auts2* and *Foxp2*, which are also transcriptional targets of AR, were also reduced in the hippocampus of male offspring prenatally exposed to BPA but not in females [13]. The correlation analysis revealed sex-specific relationships between the expression levels of AR and its targets (i.e., *Auts2, Chd2, Esrrb, Foxp2, Kmt2c,* and *Snap25*) (Fig. 5), suggesting that AR is involved in the sex-specific effects of BPA on the expression levels of these ASD candidate genes. Interestingly, the patterns of the correlations between the expression levels of TFs and their targets in the male offspring exposed to BPA tended to be more similar to those in the female control group than to the male control group. This finding suggests that prenatal BPA exposure may demasculinize the hippocampus of male pups through these TFs.

We then further investigated the role of AR in BPA-mediated effects on the expression of the *AUTS2*, *KMT2C*, and *SMARCC2* genes in the human neuronal cell line SH-SY5Y. This cell line was used as a model, because it is a human cell line and has been widely used as a model for neuroscience research. Moreover, our western blot analysis showed low expression of AR protein in SH-SY5Y cells stably transfected with the negative control plasmid (Additional file 4), making it suitable for studying the role of AR in the expression of ASD candidate genes in the presence or absence of BPA. We found that BPA significantly suppressed the expression levels of *AUTS2* and *SMARCC2* but enhanced *KMT2C* in cells with high AR expression when compared to cells with low AR expression. These results suggest that BPA suppresses *AUTS2* and *SMARCC2* expression and enhances *KMT2C* expression in human neuronal cells through AR. In addition to sex hormone receptors, sex chromosomes are thought to play an important role in sex differences in the brain [110]. As SH-SY5Y cells are derived from a female patient with XX chromosomes, future studies should include human neuronal cell lines with XY chromosomes for comparisons.

Notably, *AUTS2*, *SMARCC2*, and *KMT2C* are known to be ASD-candidate genes [101, 111, 112]. *AUTS2* (Autism Susceptibility Gene 2 or Activator of Transcription and Developmental Regulator) has been associated with ASD [111]. Mutations in the *Auts2* gene cause developmental delay, intellectual disability, microcephaly, and short stature [113]. Li et al. revealed that mice with low expression of *Auts2* showed hypoplasia in the dentate gyrus of the hippocampus [114]. Studies in zebrafish showed that *Auts2* gene disruptions caused an increase in cell death during the neuronal differentiation stage [115]. *SMARCC2* (SWI/SNF Related, Matrix Associated, Actin Dependent Regulator of Chromatin Subfamily C Member 2) encodes a member of the SWI/SNF family of proteins involved in transcriptional activation and repression by the chromatin remodeling process [116]. De novo variants in the *SMARCC2* gene cause intellectual disability and developmental delay and have been associated with ASD [117]. *KMT2C* (lysine methyltransferase 2C) is an autism candidate gene and downstream target gene of AR. *Kmt2c* is involved in the histone methyltransferase process that catalyzes the methyl group to the amino acid group of Lys-4' of histone H3 (H3K4) [118]. Mutations in the *Kmt2c* gene have been reported in neurodevelopmental disorders, including ASD, Kleefstra syndrome, and intellectual disability [119, 120]. Frega et al. demonstrated that the deficiency in *KMT2C*, along with other genes (i.e., *EHMT1*, *SMARCB1*, and *MDB5*), led to hyperactivity in neuronal network communication and altered the excitatory and inhibitory balance [121].

Since transcriptional targets of ASD-related TFs, including AR, were associated with synaptic functions, we further performed synaptogenesis assays and found a sex difference in the effects of prenatal BPA exposure on synaptic protein expression and synaptogenesis (Fig. 7). Prenatal BPA exposure caused an increase in the expression levels of synaptic proteins in the hippocampal tissues of male offspring but not in those of females. Notably, this finding is consistent with previous studies, which reported that the loss of *Auts2* led to increased excitatory synapses, disrupting the balance between excitatory and inhibitory synapses and causing cognitive and social communication deficits [80]. Our previous study also showed that prenatal BPA exposure reduced neuronal viability and neuronal density in the hippocampus and impaired learning/memory only in male offspring, while females were unaffected [10]. Some studies found that BPA exposure through gestational and postnatal stages reduced the total spine density in the hippocampus of male offspring [11, 12]. Moreover, BPA causes neuroinflammation by microglial activation [122], suggesting that BPA may negatively impact the microglia-mediated synaptic pruning process after birth. The effects of BPA on changes in synaptic formation and synaptic pruning processes in the offspring brain from embryonic to adult stages should be investigated further.

Differences in the expression of Ar and Esr1 in the hippocampus have also been investigated. Simerly et al. examined the distribution of *Ar* and *Esr1*-expressing neurons in the hippocampus and found that the expression of *Ar* was high, while the expression of *Esr1* was low in the CA1 and CA2/3 of the hippocampus. In the dentate gyrus, *Esr1* showed high expression, whereas the expression of *Ar* was low [123]. According to our previous study, we found that neuronal viability and neuronal density were significantly reduced in CA2/3 neurons of male pups prenatally exposed to BPA [10], suggesting that BPA might deregulate the expression of genes through AR, resulting in a reduced number of neurons in the hippocampus of male offspring. Moreover, previous studies in rodents have shown that young adult males performed better in the Morris water maze and radial arm maze than females (see reviews in [124]). When male offspring were exposed to the androgen receptor antagonist flutamide prenatally, they exhibited female-like spatial learning, suggesting that direct effects of androgen signaling are required [125]. Inasmuch as BPA is also known to be an AR antagonist similar to flutamide, it is possible that BPA reduced the learning/memory of male offspring through AR.

### Perspectives and significance

In summary, we propose that maternal BPA exposure during gestation alters the transcriptome profiles in the hippocampus of offspring and neurological functions associated with ASD through sex-dependent mechanisms (Fig. 8). In male offspring, BPA disrupts the transcriptome profiles of genes associated with ASD in the hippocampus through direct interactions with AR and other ASD-related TFs (i.e., Esr1, Egr2, Kdm5b, Sox5, Smad4, Tcf7l2, and Yy1). In females, BPA not only directly interacts with ASD-related TFs but also dysregulates the gene expression of these TFs (i.e., *Ar*, *Esr1*, *Egr2*, *Sox5*, *Smad4*, and *Tcf7l2*) in the hippocampus. Altered TF-mediated signaling, together with other mechanisms (e.g., indirect signaling, genetic/sex chromosome-linked factors, sex hormones, and epigenetic mechanisms), causes sex-specific dysregulation of ASD-linked genes in males (i.e., *Auts2*, *Foxp2*, *Kmt2c*, *Esrrb*, and *Kat6a*) and in females (i.e., *Chd2*, *Snap25*, and *Smarcc2*) which, in turn, leads to altered neurological functions associated with ASD, particularly synaptogenesis. Male-specific downregulation of certain AR target genes, including *Auts2* and *Foxp2*, can disrupt excitatory synapses in the hippocampus, causing deficits in learning/memory and other ASD-related symptoms observed in male offspring. This study leads to a better understanding of sex differences in BPA-mediated mechanisms in the offspring hippocampus, which provides a plausible explanation of the role of environmental factors, particularly endocrine-disrupting chemicals, on the male bias of ASD and may lead to the development of therapeutic targets for BPA-related ASD in the future.

## Supplementary Information

**Additional file 1**. Sex determination in neonatal rat pups by observing the distance between the external genitalia and anus.

**Additional file 2**. Compositions of the media used for primary hippocampal cell culture in this study.

**Additional file 3**. List of primers for qRT-PCR analyses.

**Additional file 4.** Western blot analysis of AR-overexpressing human neuroblastoma SH-SY5Y cells. The expression of AR protein in stably transfected cells was significantly upregulated compared to that in negative control plasmid transfected cells. The differences between the two groups were analyzed using a two-tailed Student’s t-test. A p-value < 0.05 was considered significant.

**Additional file 5**. A list of ASD-related transcription factors.

**Additional file 6**. Hypergeometric distribution analysis results between DEGs and transcriptional targets of each TF. A p-value < 0.05 was considered significant. NA = Not applicable

**Additional file 7**. Biological functions, disorders, and pathways associated with the transcriptional targets of EGR2 that were dysregulated in the female hippocampus predicted by IPA software. Statistical significance was determined using Fisher’s exact test. A p-value < 0.05 was considered significant.

**Additional file 8**. Biological functions, disorders, and pathways associated with the transcriptional targets of KDM5B that were dysregulated in the female hippocampus predicted by IPA software. Statistical significance was determined using Fisher’s exact test. A p-value < 0.05 was considered significant.

**Additional file 9**. Biological functions, disorders, and pathways associated with the transcriptional targets of KDM5B that were dysregulated in the male hippocampus predicted by IPA software. Statistical significance was determined using Fisher’s exact test. A p-value < 0.05 was considered significant.

**Additional file 10**. Biological functions, disorders, and pathways associated with the transcriptional targets of SMAD4 that were dysregulated in the male hippocampus predicted by IPA software. Statistical significance was determined using Fisher’s exact test. A p-value < 0.05 was considered significant.

**Additional file 11**. Biological functions, disorders, and pathways associated with the transcriptional targets of SOX5 that were dysregulated in the female hippocampus predicted by IPA software. Statistical significance was determined using Fisher’s exact test. A p-value < 0.05 was considered significant.

**Additional file 12**. Biological functions, disorders, and pathways associated with the transcriptional targets of SOX5 that were dysregulated in the male hippocampus predicted by IPA software. Statistical significance was determined using Fisher’s exact test. A p-value < 0.05 was considered significant.

**Additional file 13**. Biological functions, disorders, and pathways associated with the transcriptional targets of TCF7L2 that were dysregulated in both sexes predicted by IPA software. Statistical significance was determined using Fisher’s exact test. A p-value < 0.05 was considered significant

**Additional file 14**. Biological functions, disorders, and pathways associated with the transcriptional targets of YY1 that were dysregulated in the male hippocampus predicted by IPA software. Statistical significance was determined using Fisher’s exact test. A p-value < 0.05 was considered significant.

**Additional file 15**. ASD-related transcription factor expression data from The Allen Brain Atlas by In Situ Hybridization at several stages of brain development. Expression data of ASD-related transcription factors in developing hippocampus-related areas (rostral secondary prosencephalon; RSP and telencephalic vesicle; Tel). All data processing was performed by R software, and raw expression data were calculated from the sum of expressing pixel intensities divided by the total number of pixels that intersect. NA = Not applicable.

**Additional file 16**. ASD-related TFs and their transcriptional targets were selected for qRT-PCR analysis.

**Additional file 17**. Expression of estrogen receptors in pAR-overexpressing neuroblastoma SH-SY5Y cell line.

**Additional file 18**. Previously published BPA transcriptome studies obtained from NCBI GEO DataSets were used to reanalyze BPA-responsive genes.

**Additional file 19**. List of differentially expressed genes in the hippocampus of rodent offspring prenatally/perinatally exposed to BPA from other BPA studies.

**Additional file 20**. Hypergeometric distribution analysis results between DEGs in other independent BPA studies and the transcriptional target of ASD-related transcription factors. A p-value < 0.05 was considered significant. NA = Not applicable.

## Data Availability

The transcriptome profiling data used in this study have been deposited in the NCBI GEO data set database (GSE140298).
