# Supplementary material for: Investigation of autism-related transcription factors underlying sex differences in the effects of bisphenol A on transcriptome profiles and synaptogenesis in the offspring hippocampus
Source: Biol Sex Differ. 2023 Feb 20;14:8. doi: 10.1186/s13293-023-00496-w (PMC9940328; doi:10.1186/s13293-023-00496-w)
Supplement: Supplementary file 1 — Additional file 1. Sex determination in neonatal rat pups by observing the distance between the external genitalia and anus. [file 13293_2023_496_MOESM1_ESM.docx]

**Additional file 10. Biological functions, disorders, and pathways associated with the transcriptional targets of SMAD4 that were dysregulated in the male hippocampus predicted by IPA software.** Statistical significance was determined using Fisher’s exact test. A p-value < 0.05 was considered significant.

| **Diseases or Functions** | **P-values** | **Number of genes** |
| --- | --- | --- |
| Autism spectrum disorder or intellectual disability | 6.32E-19 | 64 |
| Mental retardation | 1.44E-16 | 54 |
| Neurodevelopmental disorder | 1.54E-11 | 35 |
| Pervasive developmental disorder | 2.97E-10 | 25 |
| Global developmental delay | 1.77E-09 | 21 |
| **Nervous system and development** |  |  |
| Development of neurons | 1.16E-10 | 50 |
| Morphogenesis of neurons | 2.25E-09 | 40 |
| Neuritogenesis | 5.20E-09 | 39 |
| Excitatory postsynaptic potential | 6.71E-05 | 11 |
| **Behavior** |  |  |
| Short-term memory | 1.25E-04 | 6 |
| Anxiety | 1.45E-04 | 14 |
