# Supplementary material for: Investigation of autism-related transcription factors underlying sex differences in the effects of bisphenol A on transcriptome profiles and synaptogenesis in the offspring hippocampus
Source: Biol Sex Differ. 2023 Feb 20;14:8. doi: 10.1186/s13293-023-00496-w (PMC9940328; doi:10.1186/s13293-023-00496-w)
Supplement: Supplementary file 2 — Additional file 2. Compositions of the media used for primary hippocampal cell culture in this study. [file 13293_2023_496_MOESM2_ESM.docx]

**Additional file 11. Biological functions, disorders, and pathways associated with the transcriptional targets of SOX5 that were dysregulated in the female hippocampus predicted by IPA software.** Statistical significance was determined using Fisher’s exact test. A p-value < 0.05 was considered significant.

| **Diseases or Functions** | **P-values** | **Number of genes** |
| --- | --- | --- |
| Congenital encephalopathy | 1.54E-05 | 11 |
| Congenital neurological disorder | 1.31E-04 | 12 |
| Hypoplasia of brain | 5.14E-04 | 4 |
| Hypoplasia of endocrine gland | 6.20E-04 | 3 |
| Familial mental retardation | 6.99E-04 | 7 |
| **Nervous system and development** |  |  |
| Formation of brain | 5.16E-09 | 14 |
| Development of central nervous system | 2.06E-08 | 15 |
| Neuritogenesis | 7.75E-08 | 14 |
| Proliferation of neuronal cells | 2.86E-05 | 10 |
| **Behavior** |  |  |
| Cognition | 5.49E-04 | 8 |
