# Supplementary material for: Investigation of autism-related transcription factors underlying sex differences in the effects of bisphenol A on transcriptome profiles and synaptogenesis in the offspring hippocampus
Source: Biol Sex Differ. 2023 Feb 20;14:8. doi: 10.1186/s13293-023-00496-w (PMC9940328; doi:10.1186/s13293-023-00496-w)
Supplement: Supplementary file 3 — Additional file 3. List of primers for qRT-PCR analyses. [file 13293_2023_496_MOESM3_ESM.docx]

**Additional file 12. Biological functions, disorders, and pathways associated with the transcriptional targets of SOX5 that were dysregulated in the male hippocampus predicted by IPA software.** Statistical significance was determined using Fisher’s exact test. A p-value < 0.05 was considered significant.

| **Diseases or Functions** | **P-values** | **Number of genes** |
| --- | --- | --- |
| Mental retardation | 3.10E-08 | 13 |
| Syndromic developmental delay and intellectual disability | 1.15E-04 | 3 |
| Global developmental delay | 2.10E-04 | 5 |
| Disorder of sex development | 1.02E-03 | 3 |
| Speech and language disorders | 1.79E-03 | 3 |
| **Nervous system and development** |  |  |
| Assembly of axon initial segments | 1.00E-05 | 2 |
| Development of central nervous system | 5.24E-04 | 8 |
| Guidance of axons | 9.24E-04 | 4 |
