# Supplementary material for: Investigation of autism-related transcription factors underlying sex differences in the effects of bisphenol A on transcriptome profiles and synaptogenesis in the offspring hippocampus
Source: Biol Sex Differ. 2023 Feb 20;14:8. doi: 10.1186/s13293-023-00496-w (PMC9940328; doi:10.1186/s13293-023-00496-w)
Supplement: Supplementary file 4 — Additional file 4. Western blot analysis of AR-overexpressing human neuroblastoma SH-SY5Y cells. The expression of AR protein in stably transfected cells was significantly upregulated compared to that in negative control plasmid transfected cells. The differences between the two groups were analyzed using a two-tailed Student’s t-test. A p-value < 0.05 was considered significant. [file 13293_2023_496_MOESM4_ESM.docx]

**Additional file 13. Biological functions, disorders, and pathways associated with the transcriptional targets of TCF7L2 that were dysregulated in both sexes predicted by IPA software.** Statistical significance was determined using Fisher’s exact test. A p-value < 0.05 was considered significant.

| **Diseases or Functions** | **P-values** | **Number of genes** |
| --- | --- | --- |
| Familial congenital malformation | 4.42E-05 | 36 |
| Disorder of stature | 1.39E-04 | 11 |
| Neural tube defect | 5.30E-04 | 7 |
| Neurodevelopmental disorder | 1.08E-03 | 13 |
| Developmental epilepsy | 1.65E-03 | 6 |
| **Nervous system and development** |  |  |
| Development of central nervous system | 3.80E-08 | 26 |
| Morphology of brain | 5.87E-07 | 21 |
| Development of neurons | 3.17E-06 | 26 |
| Neuritogenesis | 9.97E-06 | 21 |
