# Supplementary material for: Investigation of autism-related transcription factors underlying sex differences in the effects of bisphenol A on transcriptome profiles and synaptogenesis in the offspring hippocampus
Source: Biol Sex Differ. 2023 Feb 20;14:8. doi: 10.1186/s13293-023-00496-w (PMC9940328; doi:10.1186/s13293-023-00496-w)
Supplement: Supplementary file 5 — Additional file 5. A list of ASD-related transcription factors. [file 13293_2023_496_MOESM5_ESM.docx]

**Additional file 14. Biological functions, disorders, and pathways associated with the transcriptional targets of YY1 that were dysregulated in the male hippocampus predicted by IPA software.** Statistical significance was determined using Fisher’s exact test. A p-value < 0.05 was considered significant.

| **Diseases or Functions** | **P-values** | **Number of genes** |
| --- | --- | --- |
| Mental retardation | 2.04E-07 | 18 |
| Intellectual disability and congenital malformations | 1.54E-04 | 6 |
| Global developmental delay | 2.12E-04 | 7 |
| Intellectual disability and speech delay | 5.81E-04 | 2 |
| Microcephaly | 6.31E-04 | 7 |
| **Nervous system and development** |  |  |
| Synaptic transmission of cerebral cortex cells | 2.72E-03 | 3 |
| Proliferation of neuronal cells | 6.01E-03 | 9 |
| Synaptic transmission of pyramidal neurons | 7.08E-03 | 2 |
| **Behavior** |  |  |
| Auditory fear memory retrieval | 0.012 | 1 |
