# Supplementary material for: Investigation of autism-related transcription factors underlying sex differences in the effects of bisphenol A on transcriptome profiles and synaptogenesis in the offspring hippocampus
Source: Biol Sex Differ. 2023 Feb 20;14:8. doi: 10.1186/s13293-023-00496-w (PMC9940328; doi:10.1186/s13293-023-00496-w)
Supplement: Supplementary file 6 — Additional file 6. Hypergeometric distribution analysis results between DEGs and transcriptional targets of each TF. A p-value < 0.05 was considered significant. NA = Not applicable [file 13293_2023_496_MOESM6_ESM.docx]

**Additional file 15. ASD-related transcription factor expression data from The Allen Brain Atlas by In Situ Hybridization at several stages of brain development.** Expression data of ASD-related transcription factors in developing hippocampus-related areas (rostral secondary prosencephalon; RSP and telencephalic vesicle; Tel). All data processing was performed by R software, and raw expression data were calculated from the sum of expressing pixel intensities divided by the total number of pixels that intersect. NA = Not applicable

| **TFs** | **Age** | **Sex** | **Theiler Stage** | **RSP (rostral secondary prosencephalon)** | | **Tel (telencephalic vesicle)** | |
| --- | --- | --- | --- | --- | --- | --- | --- |
|  |  |  |  | **Raw expression** | **log_2_** | **Raw expression** | **log_2_** |
| **AR** | E11.5 | Unknown | TS19 | 0.03 | -5 | 0.02 | -5.89 |
|  | E13.5 | Unknown | TS21 | 0 | -8.01 | 0.01 | -6.22 |
|  | E15.5 | M | TS24 | 0.01 | -7.64 | 0.01 | -6.09 |
|  | E18.5 | M | TS26 | 0.04 | -4.52 | 0.02 | -5.48 |
|  | P4 | M |  | 0.07 | -3.88 | 0.05 | -4.26 |
|  | P14 | M |  | 0.27 | -1.9 | 0.15 | -2.75 |
|  | P28 | M |  | 0.26 | -1.94 | 0.1 | -3.4 |
| **ESR1** | E11.5 | Unknown | TS20 | 0 | -7.87 | 0.01 | -7.3 |
|  | E13.5 | Unknown | TS21 | 0.04 | -4.67 | 0.09 | -3.43 |
|  | E15.5 | M | TS24 | 0.05 | -4.2 | 0.02 | -5.81 |
|  | E18.5 | M | TS26 | 0.26 | -1.93 | 0.22 | -2.15 |
|  | P4 | M |  | 0.1 | -3.28 | 0.05 | -4.29 |
|  | P14 | M |  | 0.6 | -0.73 | 0.07 | -3.9 |
|  | P28 | M |  | 0.68 | -0.56 | 0.07 | -3.91 |
| **SMAD4** | E11.5 | Unknown | TS19 | 1.87 | 0.9 | 2.82 | 1.5 |
|  | E13.5 | Unknown | TS21 | NA | NA | 3.69 | 1.88 |
|  | E15.5 | M | TS24 | 0.23 | -2.12 | 0.58 | -0.77 |
|  | E18.5 | M | TS26 | 0.36 | -1.46 | 0.57 | -0.81 |
|  | P4 | M |  | 0.1 | -3.28 | 0.24 | -2.06 |
|  | P14 | M |  | 0.79 | -0.34 | 2.57 | 1.36 |
|  | P28 | M |  | 0.33 | -1.61 | 0.72 | -0.47 |
| **KDM5B** | E11.5 | Unknown | TS19 | 4.23 | 2.08 | 3.09 | 1.63 |
|  | E13.5 | Unknown | TS21 | 3.75 | 1.91 | 5.35 | 2.42 |
|  | E15.5 | M | TS24 | 7.48 | 2.9 | 7.06 | 2.82 |
|  | E18.5 | M | TS26 | 2.26 | 1.18 | 2.81 | 1.49 |
|  | P4 | M |  | 0.72 | -0.47 | 1.88 | 0.91 |
|  | P14 | M |  | 11.56 | 3.53 | 15.17 | 3.92 |
|  | P28 | M |  | 5.28 | 2.4 | 9.6 | 3.26 |
| **EGR2** | E11.5 | Unknown | TS19 | 0.92 | -0.12 | 3.33 | 1.74 |
|  | E13.5 | Unknown | TS21 | 4.95 | 2.31 | 5.5 | 2.46 |
|  | E15.5 | M | TS24 | 0.16 | -2.65 | 0.13 | -2.92 |
|  | E18.5 | M | TS26 | 1.18 | 0.24 | 0.76 | -0.39 |
|  | P4 | M |  | 1.11 | 0.15 | 0.78 | -0.35 |
|  | P14 | M |  | 0.2 | -2.29 | 1.61 | 0.68 |
|  | P28 | M |  | 0.82 | -0.28 | 3.62 | 1.86 |
| **YY1** | E11.5 | Unknown | TS19 | NA | NA | NA | NA |
|  | E13.5 | Unknown | TS21 | NA | NA | NA | NA |
|  | E15.5 | M | TS24 | NA | NA | NA | NA |
|  | E18.5 | M | TS26 | NA | NA | NA | NA |
|  | P4 | M |  | NA | NA | NA | NA |
|  | P14 | M |  | NA | NA | NA | NA |
|  | P28 | M |  | NA | NA | NA | NA |
| **SOX5** | E11.5 | Unknown | TS19 | 1.64 | 0.71 | 2.02 | 1.02 |
|  | E13.5 | Unknown | TS21 | 0.99 | -0.02 | 4.7 | 2.23 |
|  | E15.5 | M | TS24 | 0.46 | -1.13 | 7.27 | 2.86 |
|  | E18.5 | M | TS26 | 0.07 | -3.88 | 1.61 | 0.69 |
|  | P4 | M |  | 4.93 | 2.3 | 9.24 | 3.21 |
|  | P14 | M |  | 1.35 | 0.44 | 3.36 | 1.75 |
|  | P28 | M |  | 1.35 | 0.43 | 3.73 | 1.9 |
| **TCF7L2** | E11.5 | Unknown | TS19 | 0.34 | -1.55 | 0.41 | -1.27 |
|  | E13.5 | Unknown | TS21 | 1.04 | 0.06 | 0.33 | -1.58 |
|  | E15.5 | M | TS24 | 0.54 | -0.89 | 0.82 | -0.29 |
|  | E18.5 | M | TS26 | 0.56 | -0.82 | 0.39 | -1.36 |
|  | P4 | M |  | 0.64 | -0.64 | 0.51 | -0.96 |
|  | P14 | M |  | 2.31 | 1.21 | 1.83 | 0.87 |
|  | P28 | M |  | 0.81 | -0.3 | 0.6 | -0.74 |
