# Supplementary material for: Investigation of autism-related transcription factors underlying sex differences in the effects of bisphenol A on transcriptome profiles and synaptogenesis in the offspring hippocampus
Source: Biol Sex Differ. 2023 Feb 20;14:8. doi: 10.1186/s13293-023-00496-w (PMC9940328; doi:10.1186/s13293-023-00496-w)
Supplement: Supplementary file 8 — Additional file 8. Biological functions, disorders, and pathways associated with the transcriptional targets of KDM5B that were dysregulated in the female hippocampus predicted by IPA software. Statistical significance was determined using Fisher’s exact test. A p-value < 0.05 was considered significant. [file 13293_2023_496_MOESM8_ESM.docx]

**Additional file 17. Expression of estrogen receptors in pAR-overexpressing neuroblastoma SH-SY5Y cell line.**
