# Supplementary material for: Investigation of autism-related transcription factors underlying sex differences in the effects of bisphenol A on transcriptome profiles and synaptogenesis in the offspring hippocampus
Source: Biol Sex Differ. 2023 Feb 20;14:8. doi: 10.1186/s13293-023-00496-w (PMC9940328; doi:10.1186/s13293-023-00496-w)
Supplement: Supplementary file 11 — Additional file 11. Biological functions, disorders, and pathways associated with the transcriptional targets of SOX5 that were dysregulated in the female hippocampus predicted by IPA software. Statistical significance was determined using Fisher’s exact test. A p-value < 0.05 was considered significant. [file 13293_2023_496_MOESM11_ESM.docx]

**Additional file 1. Sex determination in neonatal rat pups by observing the distance between the external genitalia and anus.**

| **Female pup** | **Male pup** |
| --- | --- |
| 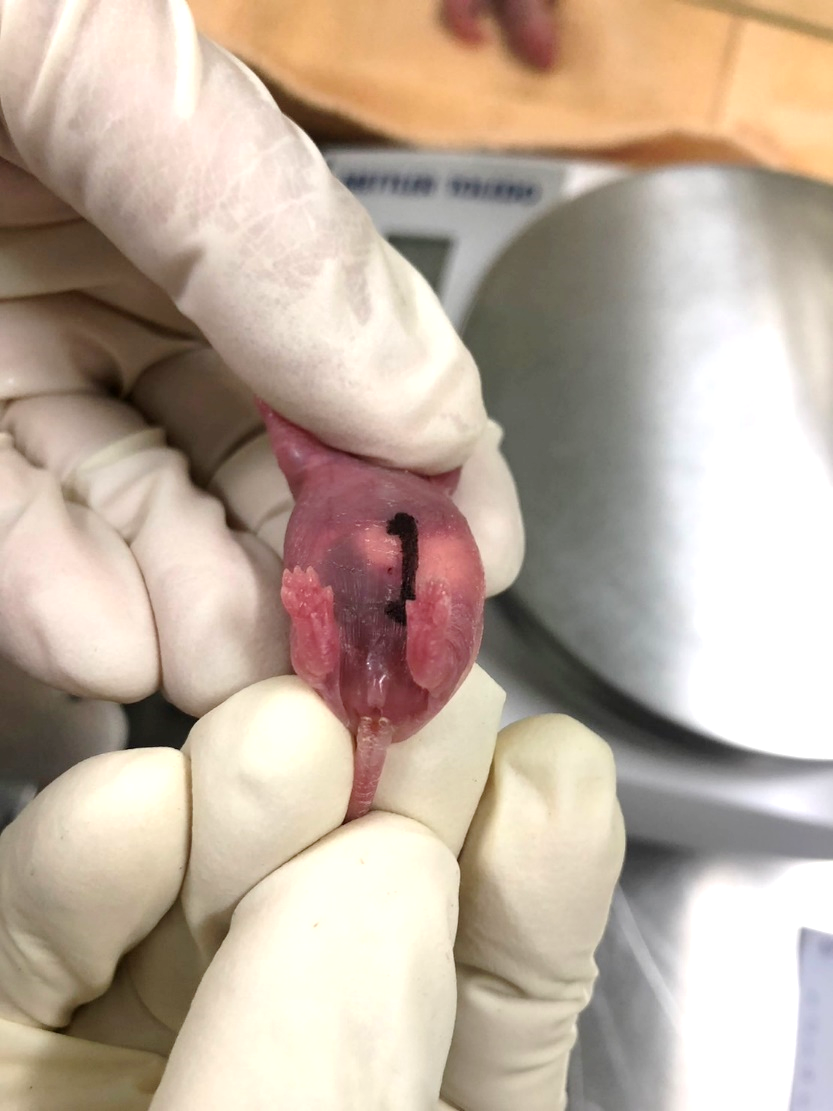 External genitalia  Anus 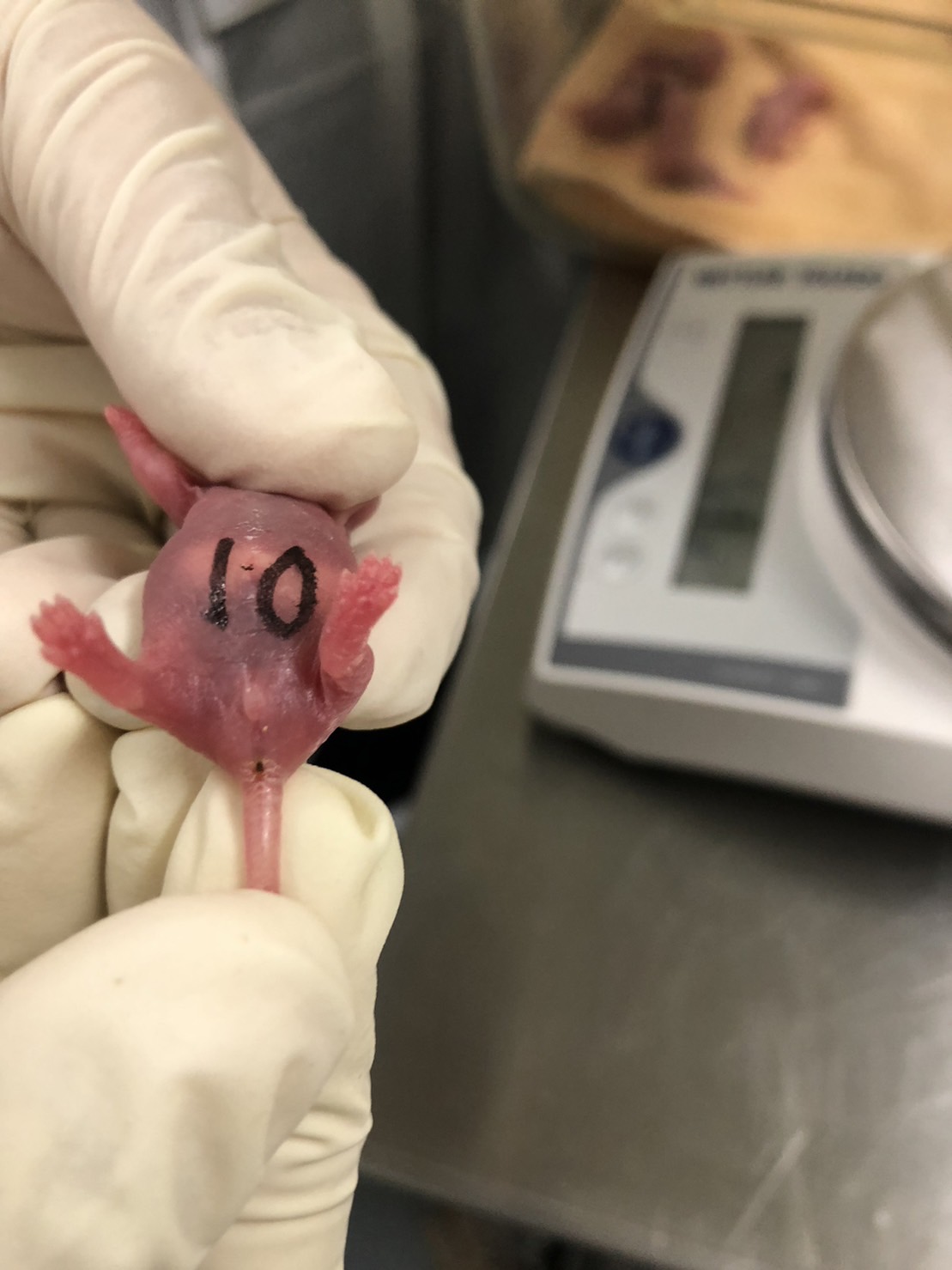 External genitalia  Anus |  |
