# Supplementary material for: Investigation of autism-related transcription factors underlying sex differences in the effects of bisphenol A on transcriptome profiles and synaptogenesis in the offspring hippocampus
Source: Biol Sex Differ. 2023 Feb 20;14:8. doi: 10.1186/s13293-023-00496-w (PMC9940328; doi:10.1186/s13293-023-00496-w)
Supplement: Supplementary file 12 — Additional file 12. Biological functions, disorders, and pathways associated with the transcriptional targets of SOX5 that were dysregulated in the male hippocampus predicted by IPA software. Statistical significance was determined using Fisher’s exact test. A p-value < 0.05 was considered significant. [file 13293_2023_496_MOESM12_ESM.docx]

**Additional file 20. Hypergeometric distribution analysis results between DEGs in other independent BPA studies and the transcriptional target of ASD-related transcription factors.** A p-value < 0.05 was considered significant. NA = Not applicable

**Study No.1** Bisphenol a exposure decreases learning ability through the suppression of mitochondrial oxidative phosphorylation in the hippocampus of male mice

Comparison: BPA 0.01 mg/L vs vehicle control

|  | **Male (DEGs = 828)** | | | **Female** | | | **Both sexes** | | |
| --- | --- | --- | --- | --- | --- | --- | --- | --- | --- |
| **TFs** | **TRANSFAC Curated**  p-value (# Overlapping genes) | **TRANSFAC Predicted**  p-value (# Overlapping genes) | **CHEA**  p-value (# Overlapping genes) | **TRANSFAC Curated**  p-value (# Overlapping genes) | **TRANSFAC Predicted**  p-value (# Overlapping genes) | **CHEA**  p-value (# Overlapping genes) | **TRANSFAC Curated**  p-value (# Overlapping genes) | **TRANSFAC Predicted**  p-value (# Overlapping genes) | **CHEA**  p-value (# Overlapping genes) |
| **AR** | **0.047** (41/755) | NA | 0.964 (237/6,279) | NA | NA | NA | NA | NA | NA |
| **ESR1** | **0.001** (34/465) | NA | 0.529 (93/2,253) | NA | NA | NA | NA | NA | NA |
| **EGR2** | 0.263 (10/190) | NA | NA | NA | NA | NA | NA | NA | NA |
| **KDM5B** | NA | NA | 0.941 (131/3,555) | NA | NA | NA | NA | NA | NA |
| **SOX5** | 0.629 (10/258) | NA | NA | NA | NA | NA | NA | NA | NA |
| **SMAD4** | **0.033** (16/233) | **0.022** (141/2,904) | **0.025** (207/4,430) | NA | NA | NA | NA | NA | NA |
| **TCF7L2** | NA | NA | 0.068 (31/565) | NA | NA | NA | NA | NA | NA |
| **YY1** | **0.007** (45/744) | **0.003** (89/1,602) | **0.025** (122/2,488) | NA | NA | NA | NA | NA | NA |

**Study No.1** Bisphenol a exposure decreases learning ability through the suppression of mitochondrial oxidative phosphorylation in the hippocampus of male mice

Comparison: BPA 0.1 mg/L vs vehicle control

|  | **Male (DEGs = 33)** | | | **Female** | | | **Both sexes** | | |
| --- | --- | --- | --- | --- | --- | --- | --- | --- | --- |
| **TFs** | **TRANSFAC Curated**  p-value (# Overlapping genes) | **TRANSFAC Predicted**  p-value (# Overlapping genes) | **CHEA**  p-value (# Overlapping genes) | **TRANSFAC Curated**  p-value (# Overlapping genes) | **TRANSFAC Predicted**  p-value (# Overlapping genes) | **CHEA**  p-value (# Overlapping genes) | **TRANSFAC Curated**  p-value (# Overlapping genes) | **TRANSFAC Predicted**  p-value (# Overlapping genes) | **CHEA**  p-value (# Overlapping genes) |
| **AR** | 1 (0/755) | NA | 0.971 (6/6,279) | NA | NA | NA | NA | NA | NA |
| **ESR1** | 0.540 (1/465) | NA | 0.899 (2/2,253) | NA | NA | NA | NA | NA | NA |
| **EGR2** | 1 (0/190) | NA | NA | NA | NA | NA | NA | NA | NA |
| **KDM5B** | NA | NA | 0.949 (3/3,555) | NA | NA | NA | NA | NA | NA |
| **SOX5** | 1 (0/258) | NA | NA | NA | NA | NA | NA | NA | NA |
| **SMAD4** | 0.321 (1/233) | 0.963 (2/2,904) | 0.885 (5/4,430) | NA | NA | NA | NA | NA | NA |
| **TCF7L2** | NA | NA | 0.612 (1/565) | NA | NA | NA | NA | NA | NA |
| **YY1** | 1 (0/744) | 0.499 (3/1,602) | 0.796 (3/2,488) | NA | NA | NA | NA | NA | NA |

**Study No.1** Bisphenol a exposure decreases learning ability through the suppression of mitochondrial oxidative phosphorylation in the hippocampus of male mice

Comparison: BPA 1 mg/L vs vehicle control

|  | **Male (DEGs = 24)** | | | **Female** | | | **Both sexes** | | |
| --- | --- | --- | --- | --- | --- | --- | --- | --- | --- |
| **TFs** | **TRANSFAC Curated**  p-value (# Overlapping genes) | **TRANSFAC Predicted**  p-value (# Overlapping genes) | **CHEA**  p-value (# Overlapping genes) | **TRANSFAC Curated**  p-value (# Overlapping genes) | **TRANSFAC Predicted**  p-value (# Overlapping genes) | **CHEA**  p-value (# Overlapping genes) | **TRANSFAC Curated**  p-value (# Overlapping genes) | **TRANSFAC Predicted**  p-value (# Overlapping genes) | **CHEA**  p-value (# Overlapping genes) |
| **AR** | 1 (0/755) | NA | 0.999 (3/6,279) | NA | NA | NA | NA | NA | NA |
| **ESR1** | 1 (0/465) | NA | 0.981 (1/2,253) | NA | NA | NA | NA | NA | NA |
| **EGR2** | 1 (0/190) | NA | NA | NA | NA | NA | NA | NA | NA |
| **KDM5B** | NA | NA | 0.987 (2/3,555) | NA | NA | NA | NA | NA | NA |
| **SOX5** | 1 (0/258) | NA | NA | NA | NA | NA | NA | NA | NA |
| **SMAD4** | 1 (0/233) | 0.994 (1/2,904) | 0.997 (2/4,430) | NA | NA | NA | NA | NA | NA |
| **TCF7L2** | NA | NA | 1 (0/565) | NA | NA | NA | NA | NA | NA |
| **YY1** | 1 (0/744) | 1 (0/1,602) | 1 (0/2,488) | NA | NA | NA | NA | NA | NA |

**Study No.2** Converging Effects of Three Different Endocrine Disrupters on Sox and Pou Gene Expression in Developing Rat Hippocampus: Possible Role of microRNA in Sex Differences

Comparison: BPA 5,000 µg/kg·BW vs vehicle control

|  | **Male (DEGs = 834)** | | | **Female (DEGs = 551)** | | | **Both sexes (DEGs = 630)** | | |
| --- | --- | --- | --- | --- | --- | --- | --- | --- | --- |
| **TFs** | **TRANSFAC Curated**  p-value (# Overlapping genes) | **TRANSFAC Predicted**  p-value (# Overlapping genes) | **CHEA**  p-value (# Overlapping genes) | **TRANSFAC Curated**  p-value (# Overlapping genes) | **TRANSFAC Predicted**  p-value (# Overlapping genes) | **CHEA**  p-value (# Overlapping genes) | **TRANSFAC Curated**  p-value (# Overlapping genes) | **TRANSFAC Predicted**  p-value (# Overlapping genes) | **CHEA**  p-value (# Overlapping genes) |
| **AR** | 0.956 (29/755) | NA | 0.999 (263/6,279) | 0.460 (26/755) | NA | 0.999 (145/6,279) | 0.740 (26/755) | NA | 0.999 (175/6,279) |
| **ESR1** | 0.989 (14/465) | NA | 0.969 (97/2,253) | 0.383 (17/465) | NA | 0.624 (73/2,253) | 0.985 (10/465) | NA | 0.988 (68/2,253) |
| **EGR2** | 0.853 (7/190) | NA | NA | 0.954 (3/190) | NA | NA | 0.594 (7/190) | NA | NA |
| **KDM5B** | NA | NA | 0.971 (159/3,555) | NA | NA | 0.999 (79/3,555) | NA | NA | 0.477 (137/3,555) |
| **SOX5** | 0.437 (14/258) | NA | NA | 0.155 (12/258) | NA | NA | 0.191 (13/258) | NA | NA |
| **SMAD4** | 0.403 (13/233) | 0.953 (130/2,904) | 0.983 (199/4,430) | 0.793 (6/233) | 0.880 (87/2,904) | 0.977 (128/4,430) | 0.672 (8/233) | 0.995 (88/2,904) | 0.999 (133/4,430) |
| **TCF7L2** | NA | NA | 0.997 (16/565) | NA | NA | 0.188 (23/565) | NA | NA | 0.971 (14/565) |
| **YY1** | 0.507 (38/744) | 0.973 (66/1,602) | 0.962 (109/2,488) | 0.965 (17/744) | 0.930 (44/1,602) | 0.999 (58/2,488) | 0.162 (34/744) | 0.998 (41/1,602) | 0.985 (77/2,488) |

**Study No.3** Mice Exposed to Bisphenol A Exhibit Depressive-like Behavior with Neurotransmitter and Neuroactive Steroid Dysfunction

Comparison: BPA 50,000 µg/kg·BW vs vehicle control

|  | **Male (DEGs = 257)** | | | **Female** | | | **Both sexes** | | |
| --- | --- | --- | --- | --- | --- | --- | --- | --- | --- |
| **TFs** | **TRANSFAC Curated**  p-value (# Overlapping genes) | **TRANSFAC Predicted**  p-value (# Overlapping genes) | **CHEA**  p-value (# Overlapping genes) | **TRANSFAC Curated**  p-value (# Overlapping genes) | **TRANSFAC Predicted**  p-value (# Overlapping genes) | **CHEA**  p-value (# Overlapping genes) | **TRANSFAC Curated**  p-value (# Overlapping genes) | **TRANSFAC Predicted**  p-value (# Overlapping genes) | **CHEA**  p-value (# Overlapping genes) |
| **AR** | 0.726 (9/755) | NA | 0.956 (75/6,279) | NA | NA | NA | NA | NA | NA |
| **ESR1** | 0.958 (3/465) | NA | 0.764 (28/2,253) | NA | NA | NA | NA | NA | NA |
| **EGR2** | 0.744 (2/190) | NA | NA | NA | NA | NA | NA | NA | NA |
| **KDM5B** | NA | NA | 0.946 (40/3,555) | NA | NA | NA | NA | NA | NA |
| **SOX5** | 0.617 (3/258) | NA | NA | NA | NA | NA | NA | NA | NA |
| **SMAD4** | 0.631 (3/233) | 0.994 (27/2,904) | 0.498 (62/4,430) | NA | NA | NA | NA | NA | NA |
| **TCF7L2** | NA | NA | **1.31E-04** (20/565) | NA | NA | NA | NA | NA | NA |
| **YY1** | 0.816 (8/744) | 0.857 (18/1,602) | 0.641 (33/2,488) | NA | NA | NA | NA | NA | NA |

**Study No.4** Impact of Low Dose Oral Exposure to Bisphenol A (BPA) on the Neonatal Rat Hypothalamic and Hippocampal Transcriptome: A CLARITY-BPA Consortium Study

Comparison: BPA 2.5 µg/kg·BW vs vehicle control

|  | **Male (DEGs =1)** | | | **Female (DEGs =13)** | | | **Both sexes** | | |
| --- | --- | --- | --- | --- | --- | --- | --- | --- | --- |
| **TFs** | **TRANSFAC Curated**  p-value (# Overlapping genes) | **TRANSFAC Predicted**  p-value (# Overlapping genes) | **CHEA**  p-value (# Overlapping genes) | **TRANSFAC Curated**  p-value (# Overlapping genes) | **TRANSFAC Predicted**  p-value (# Overlapping genes) | **CHEA**  p-value (# Overlapping genes) | **TRANSFAC Curated**  p-value (# Overlapping genes) | **TRANSFAC Predicted**  p-value (# Overlapping genes) | **CHEA**  p-value (# Overlapping genes) |
| **AR** | 1 (0/755) | NA | 1 (0/6,279) | 0.480 (1/755) | NA | 0.949 (3/6,279) | NA | NA | NA |
| **ESR1** | 1 (0/465) | NA | 1 (0/2,253) | 1 (0/465) | NA | 0.295 (3/2,253) | NA | NA | NA |
| **EGR2** | 1 (0/190) | NA | NA | 1 (0/190) | NA | NA | NA | NA | NA |
| **KDM5B** | NA | NA | 1 (0/3,555) | NA | NA | **5.30E-04** (9/3,555) | NA | NA | NA |
| **SOX5** | 1 (0/258) | NA | NA | 1 (0/258) | NA | NA | NA | NA | NA |
| **SMAD4** | 1 (0/233) | 1 (0/2,904) | 1 (0/4,430) | 1 (0/233) | 0.457 (3/2,904) | 0.770 (3/4,430) | NA | NA | NA |
| **TCF7L2** | NA | NA | 1 (0/565) | NA | NA | 1 (0/565) | NA | NA | NA |
| **YY1** | 1 (0/744) | 1 (0/1,602) | 1 (0/2,488) | **0.022 (3/744**) | 0.760 (1/1,602) | **0.046 (5/2,488)** | NA | NA | NA |

**Study No.4** Impact of Low Dose Oral Exposure to Bisphenol A (BPA) on the Neonatal Rat Hypothalamic and Hippocampal Transcriptome: A CLARITY-BPA Consortium Study

Comparison: BPA 2,500 µg/kg·BW vs vehicle control

|  | **Male (DEGs =10)** | | | **Female** | | | **Both sexes** | | |
| --- | --- | --- | --- | --- | --- | --- | --- | --- | --- |
| **TFs** | **TRANSFAC Curated**  p-value (# Overlapping genes) | **TRANSFAC Predicted**  p-value (# Overlapping genes) | **CHEA**  p-value (# Overlapping genes) | **TRANSFAC Curated**  p-value (# Overlapping genes) | **TRANSFAC Predicted**  p-value (# Overlapping genes) | **CHEA**  p-value (# Overlapping genes) | **TRANSFAC Curated**  p-value (# Overlapping genes) | **TRANSFAC Predicted**  p-value (# Overlapping genes) | **CHEA**  p-value (# Overlapping genes) |
| **AR** | 1 (0/755) | NA | 0.392 (5/6,279) | NA | NA | NA | NA | NA | NA |
| **ESR1** | 1 (0/465) | NA | **0.047** (4/2,253) | NA | NA | NA | NA | NA | NA |
| **EGR2** | 1 (0/190) | NA | NA | NA | NA | NA | NA | NA | NA |
| **KDM5B** | NA | NA | 0.184 (4/3,555) | NA | NA | NA | NA | NA | NA |
| **SOX5** | 1 (0/258) | NA | NA | NA | NA | NA | NA | NA | NA |
| **SMAD4** | 1 (0/233) | 1 (0/2,904) | 0.833 (2/4,430) | NA | NA | NA | NA | NA | NA |
| **TCF7L2** | NA | NA | 1 (0/565) | NA | NA | NA | NA | NA | NA |
| **YY1** | 0.082 (2/744) | 0.669 (1/1,602) | 0.830 (1/2,488) | NA | NA | NA | NA | NA | NA |
