# Supplementary material for: Investigation of autism-related transcription factors underlying sex differences in the effects of bisphenol A on transcriptome profiles and synaptogenesis in the offspring hippocampus
Source: Biol Sex Differ. 2023 Feb 20;14:8. doi: 10.1186/s13293-023-00496-w (PMC9940328; doi:10.1186/s13293-023-00496-w)

**Additional file 4. Western blot analysis of AR-overexpressing human neuroblastoma SH-SY5Y cells.** The expression of AR protein in stably transfected cells was significantly upregulated compared to that in negative control plasmid transfected cells. The differences between the two groups were analyzed using a two-tailed Student’s t-test. A p-value < 0.05 was considered significant.


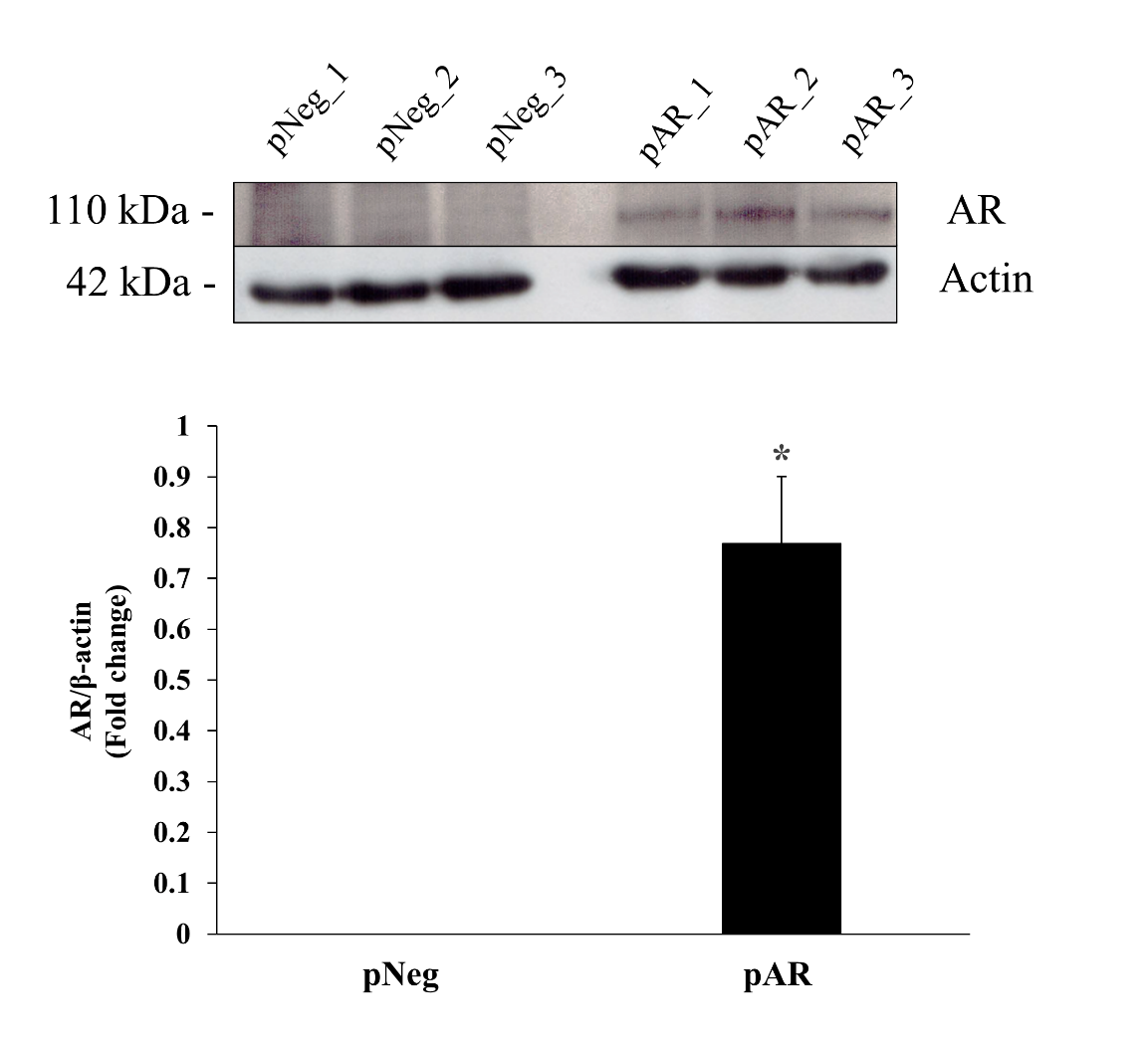

Supplement: Supplementary file 15 — Additional file 15. ASD-related transcription factor expression data from The Allen Brain Atlas by In Situ Hybridization at several stages of brain development. Expression data of ASD-related transcription factors in developing hippocampus-related areas (rostral secondary prosencephalon; RSP and telencephalic vesicle; Tel). All data processing was performed by R software, and raw expression data were calculated from the sum of expressing pixel intensities divided by the total number of pixels that intersect. NA = Not applicable. [file 13293_2023_496_MOESM15_ESM.docx]
