# Supplementary material for: Investigation of autism-related transcription factors underlying sex differences in the effects of bisphenol A on transcriptome profiles and synaptogenesis in the offspring hippocampus
Source: Biol Sex Differ. 2023 Feb 20;14:8. doi: 10.1186/s13293-023-00496-w (PMC9940328; doi:10.1186/s13293-023-00496-w)
Supplement: Supplementary file 18 — Additional file 18. Previously published BPA transcriptome studies obtained from NCBI GEO DataSets were used to reanalyze BPA-responsive genes. [file 13293_2023_496_MOESM18_ESM.docx]

**Additional file 7. Biological functions, disorders, and pathways associated with the transcriptional targets of EGR2 that were dysregulated in the female hippocampus predicted by IPA software.** Statistical significance was determined using Fisher’s exact test. A p-value < 0.05 was considered significant.

| **Diseases or Functions** | **P-values** | **Number of genes** |
| --- | --- | --- |
| Abnormal morphology of forelimb bud | 2.00E-05 | 2 |
| Hypoplasia of cells | 7.41E-05 | 3 |
| Progressive muscular dystrophy | 8.21E-05 | 5 |
| Abnormal morphology of embryonic tissue | 6.99E-04 | 7 |
| **Nervous system and development** |  |  |
| Abnormal morphology of nervous system | 3.38E-07 | 13 |
| Abnormal morphology of neurons | 1.09E-06 | 10 |
| Cell viability of neurons | 2.93E-05 | 6 |
| Loss of neurons | 7.59E-05 | 5 |
| **Behavior** |  |  |
| Learning | 4.03E-06 | 9 |
| Memory | 1.27E-05 | 7 |
