# Supplementary material for: Investigation of autism-related transcription factors underlying sex differences in the effects of bisphenol A on transcriptome profiles and synaptogenesis in the offspring hippocampus
Source: Biol Sex Differ. 2023 Feb 20;14:8. doi: 10.1186/s13293-023-00496-w (PMC9940328; doi:10.1186/s13293-023-00496-w)
Supplement: Supplementary file 19 — Additional file 19. List of differentially expressed genes in the hippocampus of rodent offspring prenatally/perinatally exposed to BPA from other BPA studies. [file 13293_2023_496_MOESM19_ESM.docx]

**Additional file 8. Biological functions, disorders, and pathways associated with the transcriptional targets of KDM5B that were dysregulated in the female hippocampus predicted by IPA software.** Statistical significance was determined using Fisher’s exact test. A p-value < 0.05 was considered significant.

| **Diseases or Functions** | **P-values** | **Number of genes** |
| --- | --- | --- |
| Microcephaly | 4.99E-16 | 45 |
| Congenital neurological disorder | 4.69E-15 | 98 |
| Autism or intellectual disability | 8.98E-15 | 84 |
| Growth failure or short stature | 1.49E-14 | 84 |
| Abnormal morphology of embryo | 1.60E-12 | 67 |
| **Nervous system and development** |  |  |
| Morphology of nervous system | 5.33E-11 | 96 |
| Formation of brain | 3.93E-10 | 58 |
| Development of neurons | 6.35E-09 | 78 |
| Growth of neurites | 1.12E-08 | 50 |
| **Behavior** |  |  |
| Cognition | 3.79E-05 | 32 |
| Learning | 3.90E-05 | 30 |
