# Supplementary material for: Investigation of autism-related transcription factors underlying sex differences in the effects of bisphenol A on transcriptome profiles and synaptogenesis in the offspring hippocampus
Source: Biol Sex Differ. 2023 Feb 20;14:8. doi: 10.1186/s13293-023-00496-w (PMC9940328; doi:10.1186/s13293-023-00496-w)
Supplement: Supplementary file 20 — Additional file 20. Hypergeometric distribution analysis results between DEGs in other independent BPA studies and the transcriptional target of ASD-related transcription factors. A p-value < 0.05 was considered significant. NA = Not applicable. [file 13293_2023_496_MOESM20_ESM.docx]

**Additional file 9. Biological functions, disorders, and pathways associated with the transcriptional targets of KDM5B that were dysregulated in the male hippocampus predicted by IPA software.** Statistical significance was determined using Fisher’s exact test. A p-value < 0.05 was considered significant.

| **Diseases or Functions** | **P-values** | **Number of genes** |
| --- | --- | --- |
| Nonspecific mental retardation | 7.40E-24 | 52 |
| Autism spectrum disorder or intellectual disability | 7.45E-24 | 85 |
| Neurodevelopmental disorder | 2.10E-15 | 48 |
| Global developmental delay | 1.62E-13 | 30 |
| Microcephaly | 3.34E-12 | 31 |
| Pervasive developmental disorder | 1.55E-07 | 25 |
| **Nervous system and development** |  |  |
| Morphology of neurites | 1.55E-06 | 25 |
| Neuritogenesis | 1.57E-05 | 39 |
| Proliferation of neuronal cells | 2.55E-05 | 34 |
| **Behavior** |  |  |
| Cognition | 3.79E-05 | 32 |
| Learning | 3.90E-05 | 30 |
